# Contemporary Adjuvant Chemotherapy for Intraductal Papillary Mucinous Neoplasms

**DOI:** 10.1001/jamanetworkopen.2026.3688

**Published:** 2026-03-27

**Authors:** James Lucocq, Beate Haugk, Steven White, Giovanni Marchegiani, Marcus Holmberg, Alessandro Zerbi, Michele Pagnanelli, Zipeng Lu, Yosuke Inoue, Munseok Choi, Chang Moo Kang, Brendan Visser, Lieke Corpelijn, Bodil Andersson, Paulina Bereza-Carlson, Bergthor Björnsson, Johanna Wennerblom, Knut Jørgen Labori, Brian K. P. Goh, Vera Hartman, Syed Ahmad, Sameer Patel, Frederik Berrevoet, Kim Mortensen, Keith Roberts, Fabio Ausania, Calum Lynch, Amit Gupta, Claude Bertrand, Joerg Kleeff, Pietro Addeo, Ruben Bellotti, Zhi Ven Fong, Helmut Friess, Jonathan Koea, Dhanny Gomez, Alex Gordon-Weeks, Cataldo Doria, Georgios Tzimas, Oskar Franklin, Christopher Månsson, Santiago Sánchez Cabús, Ricky Harminder Bhogal, Samir Pathak, Kim Christin Honselmann, Anita Balakrishnan, Ewen Harrison, Kulbir Mann, Anthony J. Gill, Anubhav Mittal, Jas Samra, Tejinderjit Athwal, Benjamin Loveday, Catherine Teh, Neil Bhardwaj, Krishna Menon, Prabin Thapa, Ippei Matsumoto, Nigel Jamieson, Vinciane Rebours, Clemence Descourvieres, Brian Davidson, Kjetil Soreide, Sanjay Pandanaboyana

**Affiliations:** 1 Department of Hepatopancreatobiliary Surgery, Royal Infirmary of Edinburgh, United Kingdom; 2 Department of Hepato-Pancreato-Biliary and Transplant Surgery, Freeman Hospital, Newcastle, United Kingdom; 3 Hepatopancreatobiliary and Liver Transplant Surgery, Department of Surgery, Oncology and Gastroenterology, University of Padova, Italy; 4 Department of Clinical Science, Intervention and Technology, Karolinska Institutet, Sweden; 5 Department of Biomedical Sciences, Humanitas University, Pancreatic Unit, Istituto di Ricovero e Cura a Carattere Scientifico Humanitas Research Hospital, Milan Italy; 6 Pancreas Centre, The First Affiliated Hospital with Nanjing Medical University, Nanjing, China; 7 Cancer Institute Hospital of Japanese Foundation for Cancer Research, Hepato-Biliary-Pancreatic Medicine Department, Tokyo, Japan; 8 Division of Hepatobiliary and Pancreatic Surgery, Department of Surgery, Yongin Severance Hospital, Yonsei University College of Medicine, South Korea; 9 Stanford Cancer Centre, Palo Alto, California; 10 Skane University Hospital, Lund, Sweden; 11 Department of Surgery and Clinical and Experimental Medicine, Linköping University, Linköping, Sweden; 12 Department of Surgery, The Institute of Clinical Sciences, Gothenburg, Sweden; 13 Department of Hepatopancreatobiliary Surgery, Oslo university Hospital, Norway and Institute of Clinical Medicine, University of Oslo, Norway; 14 Department of Hepatopancreatobiliary and Transplant Surgery, Singapore General Hospital & National Cancer Center, Singapore; 15 Antwerp University Hospital, Edegem, Belgium; 16 Division of Surgical Oncology, University of Cincinnati; 17 Department for General and Hepatopancreatobiliary Surgery and Liver Transplantation, Ghent University Hospital, Belgium; 18 University Hospital North Norway, Tromso, Norway; 19 University Hospitals Birmingham, United Kingdom; 20 Hepato-Pancreato-Biliary and Transplant Surgery, Hospital Clinic Barcelona, Institut d'Investigacions Biomèdiques August Pi i Sunyer, University of Barcelona, Spain; 21 All India Institute of Medical Sciences Rishikesh, India; 22 Unit of Digestive, Endocrine, and General Surgery, Centre Hospitalier Universitaire Université Catholique de Louvain, Namur-Godinne, Belgium; 23 Department of Visceral, Vascular and Endocrine Surgery, University Hospital Halle (Saale), Martin-Luther-University Halle-Wittenberg, Halle (Saale), Germany; 24 Hautepierre Hospital, Strasbourg, France; 25 Department of Visceral, Transplant and Thoracic Surgery, Medical University of Innsbruck, Innsbruck, Austria; 26 Mayo Clinic Comprehensive Cancer Center, Phoenix, Arizona; 27 Department of Surgery, Munich, Germany; 28 North Shore Hospital, Auckland, New Zealand; 29 Nottingham University Hospitals National Health Service Trust, United Kingdom; 30 Department of Hepatopancreatobiliary Surgery, Oxford, United Kingdom; 31 Capital Health Cancer Center, Pennington, New Jersey; 32 Hygeia Hospital, Athens, Greece; 33 Department to Diagnostics and Intervention, Umeå, Sweden; 34 Department of Surgical Sciences, Uppsala University, Uppsala, Sweden; 35 Hospital de la Santa Creu I Sant Pau, Barcelona, Spain; 36 The Royal Marsden Hospital and Institute for Cancer Research, London, United Kingdom; 37 St James’s University Hospital, The Leeds Teaching Hospitals, United Kingdom; 38 Department of Surgery, University Medical Center Schleswig-Holstein, Campus Luebeck, Germany; 39 Cambridge Hepatopancreatobiliary Unit, Cambridge University Hospitals NHS Foundation Trust, Cambridge, United Kingdom; 40 Royal Liverpool Hospital, United Kingdom; 41 Royal North Shore Hospital, Sydney, Australia and University of Sydney, Sydney, Australia; 42 Royal Stoke University Hospital, United Kingdom; 43 Peter MacCallum Cancer Centre, Melbourne, Victoria, Australia; 44 Makati Medical Center, Philippines; 45 Leicester General Hospital, Leicester, United Kingdom; 46 Kings College Hospital, London, United Kingdom; 47 Department of General and Hepatopancreatobiliary Surgery, Kathmandu, Nepal; 48 Kindai University Faculty of Medicine, Osaka, Japan; 49 Glasgow Royal Infirmary, National Health Service Greater Glasgow and Clyde, United Kingdom; 50 Pancreatology and Digestive Oncology Department, Beaujon Hospital, Clichy, France, Université Paris-Cité; 51 Royal Free London National Health Service Foundation Trust, United Kingdom; 52 Department of Gastrointestinal Surgery, Stavanger University Hospital, Stavanger, Norway

## Abstract

**Importance:**

Adjuvant chemotherapy regimens may be administered after pancreatic resection for adenocarcinoma arising from intraductal papillary mucinous neoplasms (A-IPMNs), although the evidence supporting their use is limited.

**Objective:**

To evaluate the survival benefit associated with contemporary adjuvant chemotherapy regimens after resection in A-IPMNs between 2017 and 2023.

**Design, Setting, and Participants:**

This retrospective cohort study was an international, multicenter study with 69 participating centers across Europe, North America, South America, and the Asia-Pacific region. Patients undergoing resection for A-IPMNs were included. Data were analyzed from May to August 2025.

**Intervention:**

Contemporary adjuvant chemotherapy regimens, such as gemcitabine-capecitabine (GemCap); 5-fluorouracil, leucovorin, oxaliplatin, and irinotecan (FOLFIRINOX), modified FOLFIRINOX (mFOLFIRINOX), and S-1.

**Main Outcome and Measure:**

Overall survival (months).

**Results:**

Among 1321 patients (median [IQR] age 70 [63 to 76] years; 713 males [54.0%] and 608 females [46.0%]), 781 patients (59.1%) received adjuvant chemotherapy, while in 181 patients (13.7%) it was omitted due to poor patient fitness. Of patients who received adjuvant chemotherapy, 568 patients (72.6%) received contemporary regimens, including GemCap (232 patients [29.7%]), FOLFIRINOX (176 patients [22.5%]), mFOLFIRINOX (71 patients [9.1%]), and S-1 (70 patients [9.0%]). The median (IQR) follow-up for the cohort was 64.2 (40.4 to 85.0) months, and the median overall survival was 73.8 months (95% CI, 66.4 to 81.9 months). After 90-day landmark analysis and exclusion of patients ineligible for chemotherapy, adjuvant chemotherapy vs no adjuvant chemotherapy (propensity score–matched populations, 243:243 patients) was not associated with improved overall survival (median, 82.3 months; 95% CI, 78.2 months to not applicable [NA] vs not reached; 95% CI, 75.3 months to NA; *P* = .58). Contemporary regimens vs no adjuvant chemotherapy (propensity score–matched populations, 309:309 patients) was not associated with longer survival, and a mean survival benefit greater than 4.2 months over 5 years was excluded (difference in restricted mean survival, 1.26 months; 95%, −1.72 to 4.24 months). Treatment outcomes did not vary by chemotherapy regimen or disease characteristic (eg, N stage or carbohydrate antigen 19-9 level).

**Conclusions and Relevance:**

In this study, contemporary adjuvant chemotherapy was not associated with improved overall survival in A-IPMNs, and a randomized clinical trial is indicated.

## Key Points

**Question:**

What is the survival benefit associated with contemporary adjuvant chemotherapy regimens after resection in adenocarcinoma arising from intraductal papillary mucinous neoplasms (A-IPMNs)?

**Findings:**

In this cohort study among 1321 patients with A-IPMNs, adjuvant chemotherapy was not associated with improved overall survival, regardless of adjuvant chemotherapy type.

**Meaning:**

Contemporary adjuvant chemotherapy was not associated with improved overall survival, and a randomized clinical trial is indicated.

## Teaser

This cohort study investigates the association of contemporary adjuvant chemotherapy regimens after resection in adenocarcinoma arising from intraductal papillary mucinous neoplasms (A-IPMNs) with overall survival.

## Introduction

Pancreatic ductal adenocarcinoma (PDAC) has a dismal prognosis and remains a considerable surgical-oncological challenge. While PDAC is the most common form of pancreatic cancer and develops de novo, some PDAC may arise from intraductal papillary mucinous neoplasms (A-IPMNs), which constitute approximately 5% to 15% of all pancreatic adenocarcinomas.^1^ Approximately 50% of patients with A-IPMNs will experience recurrence, which tends to occur within the first year after resection in most patients.^2^ A-IPMN is a heterogeneous disease, derived from different precursor epithelial subtypes and constituted by ductal or colloid invasive components, with each component type associated with distinct prognostic implications and survival outcomes.^3,4,5^ Overall, A-IPMNs have superior survival and lower recurrence rates compared with primary PDAC.^6,7^

In patients with PDAC not associated with an underlying IPMN, adjuvant chemotherapy has become the standard of care.^8,9^ Nevertheless, these trials do not include sufficient numbers of patients with A-IPMNs to conclude the efficacy of chemotherapy in A-IPMN. Current guidelines differ in their recommendations regarding adjuvant chemotherapy for IPMNs with associated invasive carcinoma.^10,11,12,13^ While some guidelines suggest offering adjuvant chemotherapy, most do not recommend it routinely due to limited evidence.

Existing research on outcomes associated with PDAC-derived chemotherapy, such as the previous Adeno-IPMN study^14^ undertaken by our group, included predominantly outdated chemotherapy regimens (eg, gemcitabine monotherapy). While this study called into question the efficacy of adjuvant chemotherapy regimens in A-IPMN, other studies have suggested a benefit in patients with elevated carbohydrate antigen 19-9 (CA-19-9) levels or node-positive disease, albeit in small cohorts with predominantly single-agent gemcitabine adjuvant chemotherapy.^15,16,17^ Existing studies, including a 2024 large, international, multicenter observational study,^15^ are limited by their inability to adjust for selection biases such as patient fitness for consideration of adjuvant chemotherapy. The primary aim of this study was to investigate the association of contemporary adjuvant chemotherapy regimens (such as gemcitabine-capecitabine [GemCap]; 5-fluorouracil, leucovorin, oxaliplatin, and irinotecan [FOLFIRINOX]; modified FOLFIRINOX [mFOLFIRINOX]; and S-1) with survival in a large cohort of patients undergoing pancreatic resection for A-IPMNs.

## Methods

Ethical approval for this cohort study was obtained from the Newcastle Joint Research Office, and each participating center gained its own ethical approval in accordance with local policy. The study was conducted according to the Declaration of Helsinki, and informed patient consent was not required based on the local ethics committee from the Newcastle Upon Tyne Hospitals National Health Service Foundation Trust and the use of the Health Research Authority tool kit. Each site had to obtain local ethics approval prior to data input. The study was conducted in accordance with the Strengthening the Reporting of Observational Studies in Epidemiology (STROBE) reporting guideline.

### Study Population

Consecutive patients undergoing pancreatic resection for A-IPMNs between 2017 and 2023 were included. This was an international, multicenter retrospective study with 69 participating centers across Europe, North America, South America, and the Asia-Pacific region. Invasive IPMNs after pancreatic resection were identified in institutional databases. Each tumor had to be associated with an underlying IPMN with an identified precursor epithelial subtype within the tumor, as per the World Health Organization definition.^18^ This allowed exclusion of patients with tubular (ductal) adenocarcinoma. Primary PDAC, PDAC with a concomitant A-IPMN, and PDAC associated with a noninvasive IPMN were not included.^18^ We also excluded 16 patients with metastatic disease, 32 patients with locally advanced disease, 12 patients with R2 resection, and 112 patients receiving neoadjuvant chemotherapy from this analysis. Additionally, we excluded 13 centers with fewer than 5 reported cases.

Each registered center appointed a primary investigator who registered patient details on the Research Electronic Data Capture (REDCap) system. No identifiable data were uploaded to REDCap, and each case was allocated a unique and secure REDCap identification number.

### Data Collection

We obtained background demographic data, including comorbid status according to the Charlson Comorbidity Index (CCI), for all patients. We also recorded preoperative data, such as radiological findings, preoperative bilirubin level (in micromoles per liter), and CA-19-9 level (U/mL). An elevated bilirubin level was defined as 20 μmol/L or higher, and a high and very high CA-19-9 level was defined as 37 to 200 U/mL and 200U/mL or greater, respectively, to ensure consistency with other studies.^15,19,20^ We recorded operative data, including operative approach and pancreatic resection type. We collected disease-specific variables, such as precursor epithelial subtype, invasive ductal component, and duct type. The final staging according to the *TNM Classification of Malignant Tumours, 8th Edition* was then recorded with the resection (R) status.^21^ R1 status was defined by a cutoff distance from the tumor to the resection margin of less than 1 mm.

We recorded adjuvant chemotherapy administration, including the type and the number of cycles. The following adjuvant chemotherapy regimens were considered contemporary regimens: GemCap, FOLFIRINOX, mFOLFIRINOX, S-1, and gemcitabine with nanoparticle albumin–bound paclitaxel (gem-nab-paclitaxel). Omission of adjuvant chemotherapy due to poor patient fitness, oncology decision, or patient preference was recorded.

### Overall Survival and Recurrence in Unadjusted Cohort

The main outcome of interest was overall survival (OS), but recurrence and disease-free survival (DFS) were also recorded. Recurrence was diagnosed by imaging with or without histology. The site of recurrence was classified as locoregional (pancreatic, peripancreatic, or regional lymph nodes) or systemic (liver, lung, peritoneal, or other). DFS was defined as survival in the absence of recurrence. We report 1-, 3-, and 5-year OS; recurrence (including site-specific rates); and DFS rates using Kaplan-Meier curves. Patient follow-up was conducted according to the protocol of each participating center using sources such as cancer registries and general practitioner records.

Variables associated with OS were identified using univariate and multivariable Cox proportional hazards regression analysis. The most parsimonious model was derived using backward elimination, removing insignificant variables in order of least significance and guided by the Akaike information criterion (AIC). We also constructed a directed acyclic graph (DAG) to interpret findings of the multivariate model. Variables not included in the multivariate model (redundant covariates) were included in the DAG, and their association with confounders was demonstrated.

### Statistical Analysis

#### Bias-Adjusted Cohort and Adjuvant Chemotherapy Receipt

To address selection bias for adjuvant chemotherapy secondary to immortal time bias, a 90-day landmark analysis was performed, whereby we excluded 42 of 1321 patients (3.2%) who died within 90 days after the operation. Furthermore, 181 patients (13.7%) ineligible for receipt of adjuvant chemotherapy due to poor patient fitness were also excluded to mitigate against selection bias for adjuvant chemotherapy. Poor patient fitness for adjuvant chemotherapy was defined based on the assessment conducted by the multidisciplinary team. Reasons patients were considered unsuitable included frailty, poor performance status, significant comorbidities, postoperative complications, and anticipated intolerance to chemotherapy. In the bias-adjusted cohort, variables associated with adjuvant chemotherapy administration were identified using the χ^2^ test, and odds ratios (ORs) were reported.

#### Adjuvant Chemotherapy and Survival (Bias-Adjusted Cohort)

In the bias-adjusted cohort, the association between adjuvant chemotherapy and OS was determined using propensity score–matched (PSM) analysis. The adjuvant chemotherapy group (treatment group) was matched with the no adjuvant chemotherapy group (control group) for age and clinicopathological variables independently associated with OS in multivariate analysis (lesion location, operation type, invasive subtype [eg, ductal], differentiation, perineural invasion, T/N stage, and R1 margin).

The PSM used 1:1 nearest-neighbor, probit regression, and the caliper was set to 0.05. Complete-case analysis was used for propensity score matching; patients with missing covariate data were excluded from relevant analyses, and the extent of missing data is reported in eTable 1 in Supplement 1. Variable balancing was assessed using the standardized mean difference and distance-variance ratio. This analysis was repeated for contemporary adjuvant chemotherapy regimens.

We used Kaplan-Meier curves and the log-rank test to compare the survival and recurrence of treatment and control groups. Furthermore, absolute differences in survival time were quantified using restricted mean survival time (RMST) and determined from the Kaplan-Meier survival curve in PSM populations at 12, 24, 36, 48, and 60 months. The difference in RMST between treatment groups (adjuvant chemotherapy minus no adjuvant chemotherapy) was estimated and reported in months with 95% CIs (positive values indicating longer mean survival associated with adjuvant chemotherapy). We used upper and lower bounds to quantify the magnitude of survival benefit or harm that could be excluded at the .05 significance level.

A sensitivity analysis was performed by reintroducing patients considered ineligible for chemotherapy due to poor fitness. This was conducted to investigate potential spurious survival advantages and erroneous conclusions that may result from failing to account for treatment eligibility. All statistical analysis was conducted in RStudio version 2024.04.2 + 764 (RStudio). A *P* value < .05 was considered statistically significant, and 2-tailed tests were used. Data were analyzed from May to August 2025.

#### Secondary Analysis: Subgroups by Clinicopathological Features (Including Nodal Status)

The PSM analysis was repeated in individual groups categorized by age (<60, 60-69, 70-79, and ≥80 years), sex, invasive components (ductal and colloid components), T stage, N stage, and margin status. It has been suggested that adjuvant chemotherapy may be associated with improved survival in patients with involved lymph nodes and high CA-19-9 levels.^15^ Therefore, PSM was repeated in this group and derived from the bias-adjusted cohort.

#### Secondary Analysis: Chemotherapy Regimen–Specific Analyses

Individual PSMs were repeated in the bias-adjusted cohort for each individual chemotherapy regimen comparing against no adjuvant chemotherapy. Additionally, a Cox proportional hazards model was derived in the bias-adjusted cohort using variables independently associated with OS in the earlier multivariate analysis. Individual chemotherapy types were introduced as separate variables, and no adjuvant chemotherapy was treated as the reference variable.

## Results

### Cohort and Treatment Patterns

Overall, 1321 patients with A-IPMNs (median [IQR] age, 70 [63-76] years; 713 males [54.0%] and 608 females [46.0%]; male:female ratio, 1.1:1) were included (Table). Most patients (805 patients [60.9%]) had an A-IPMN in the pancreatic head, and 709 patients (53.7%) underwent a pancreatoduodenectomy, either Whipple procedure or pylorus preserved pancreatoduodenectomy (PPPD). Overall, 866 patients had stage T2 or higher disease (65.6%), 543 patients (41.0%) had positive lymph nodes, and 386 patients (29.2%) had an R1 resection. A total of 961 patients (72.7%) had a ductal tumor, and the most common precursor epithelial subtype was pancreatobiliary, occurring in 525 patients (39.7%). There were 321 patients (24.3%) with a poorly differentiated tumor, 760 patients (57.5%) with perineural invasion, and 642 patients (48.6%) with lymphovascular invasion. The preoperative CA-19-9 level was high in 344 patients (26.0%) and very high in 297 patients (22.5%), and the bilirubin level was elevated in 317 patients (24.0%). In this study, data completeness was high, with a 92% (1218 of 1321 patients had data for all variables [92.2%]) or greater completion rate for all variables included in survival analyses (range for missing data, 0 patients for age to 103 patients [7.8%] for invasive component) (eTable 1 in Supplement 1).

**Table.** Cohort Characteristics and Variables Associated With Overall Survival

Variable	Patients, No. (%) (N = 1321)	Univariate		Multivariate		Multivariate most parsimonious	
		HR (95% CI)	*P* value	HR (95% CI)	*P* value	HR (95% CI)	*P* value
Age, median (IQR), y	70 (63-76)	1.5 (1.2-1.9)^a^	.002	1.0 (0.7-1.3)^a^	.92	NA	NA
CCI score, median (IQR)	4 (3-6)	1.3 (1.1-1.5)^a^	.001	1.1 (0.9-1.5)^a^	.42	NA	NA
Location							
Head	805 (60.9)	1 [Reference]	NA	NA	NA	NA	NA
Body	225 (17.0)	1.0 (0.8-1.3)	.99	NA	NA	NA	NA
Tail	178 (13.5)	1.2 (1.0-1.6)	.06	1.4 (0.9-2.1)	.13	1.3 (1.0-1.8)	.05
Diffuse	105 (7.9)	1.4 (1.1-1.9)	.02	1.0 (0.6-1.5)	.72	NA	NA
Duct type							
Main	529 (40.0)	1 [Reference]	NA	NA	NA	NA	NA
Branch	202 (15.3)	0.85 (0.7-1.1)	.20	NA	NA	NA	NA
Mixed	484 (36.6)	1.03 (0.9-1.2)	.76	NA	NA	NA	NA
Operation							
Whipple or PPPD	709 (53.7)	1 [Reference]	NA	NA	NA	NA	NA
DP	346 (26.2)	1.3 (1.1-1.6)	.004	1.1 (0.7-1.6)	.72	NA	NA
TP	259 (19.6)	1.7 (1.4-2.1)	<.001	1.5 (1.1-2.0)	.02	1.5 (1.8-1.9)	.001
Elevated bilirubin level^b^	317 (24.0)	1.6 (1.3-1.9)	<.001	1.1 (0.8-1.5;0.46	NA	NA	NA
CA 19-9 level, U/mL^c^							
37-200	344 (26.0	1.6 (1.2-2.0)	<.001	1.9 (0.9-1.6)	.27	NA	NA
>200	297 (22.5	2.3 (1.8-2.8)	<.001	1.4 (1.0-1.9)	.05	NA	NA
T stage							
T1	427 (32.3)	1 [Reference]	NA	NA	NA	NA	NA
T2	499 (37.8)	2.4 (1.9-3.00)	<.001	1.2 (0.8-1.7)	.31	NA	NA
T3	351 (26.6)	2.6 (2.1-3.30)	<.001	1.4 (0.9-2.0)	.10	NA	NA
T4	16 (1.2)	4.0 (2.4-6.7)	<.001	2.1 (0.9-4.9)	.08	NA	NA
N stage							
N0	781 (59.1)	1 [Reference]]	NA	NA	NA	NA	NA
N1	342 (25.9)	2.2 (1.8-2.6)	<.001	1.5 (1.1-2.0)	.01	1.6 (1.2-2.0)	<.001
N2	199 (15.1)	3.4 (2.8-4.2)	<.001	2.3 (1.6-3.2)	<.001	2.2 (1.7-2.8)	<.001
Precursor subtype							
Gastric	215 (16.3)	1 [Reference]	NA	NA	NA	NA	NA
Intestinal	198 (15.0)	0.6 (0.4-0.8)	.002	0.9 (0.6-1.4)	.66	NA	NA
Pancreatobiliary	525 (39.7)	1.3 (1.0-1.6)	.05	0.8 (0.6-1.1)	.16	NA	NA
Mixed	97 (7.3)	1.2 (0.8-1.6)	.32	NA	NA	NA	NA
Invasive component							
Colloid	187 (14.2)	1 [Reference]	NA	NA	NA	NA	NA
Ductal	961 (72.7)	2.6 (2.0-3.5)	<.001	1.8 (1.1-2.7)	.011	1.8 (1.2-2.5)	.002
Differentiation							
Well	288 (21.8)	1 [Reference]	NA	NA	NA	NA	NA
Moderate	598 (45.3)	1.6 (1.3-2.0)	<.001	1.2 (0.9-1.6;.0.29	NA	NA	NA
Poor	321 (24.3)	2.7 (2.1-3.5)	<.001	1.34 (1.0-2.0)	.07	1.4 (1.1-1.7)	.003
Perineural invasion	760 (57.5)	3.1 (2.6-3.8)	<.001	2.0 (1.4-2.8)	<.001	2.0 (1.5-2.6)	<.001
Lymphovascular invasion	642 (48.6)	2.8 (2.3-3.3)	<.001	1.0 (0.8-1.4)	.85	NA	NA
R1 margin	386 (29.2)	2.2 (1.8-2.6)	<.001	1.2 (0.9-1.6)	.13	1.4 (1.1-1.7)	.004

Abbreviations: CCI, Charlson Comorbidity Index; DP, distal pancreatectomy; HR, hazard ratio; NA, not applicable; PPPD, pylorus preserved pancreatoduodenectomy; TP, total pancreatectomy.

^a^ Higher age and CCI score were defined as greater than or equal to the median compared with less than the median.

^b^ Checked in 1127 patients.

^c^ Checked in 1019 patients.

Adjuvant chemotherapy was administered in 781 patients (59.1%), including GemCap in 232 patients (29.7%), FOLFIRINOX in 176 patients (22.5%), gemcitabine monotherapy in 147 patients (18.8%), mFOLFIRINOX in 71 patients (9.1%), S-1 in 70 patients (9.0%), gem-nab-paclitaxel in 19 patients (2.4%), and other in 67 patients (8.6%). Overall, contemporary chemotherapy regimens were administered in 568 patients (72.6% of those receiving adjuvant chemotherapy).

Adjuvant chemotherapy was omitted due to poor patient fitness in 181 patients (13.7% of the total cohort), representing 33.6% of 539 patients who did not receive chemotherapy. In the remaining patients, adjuvant chemotherapy was not administered because of an oncology decision (eg, low perceived benefit) in 190 patients (35.3%) or due to patient decision in 60 patients (11.1%). In 108 patients (20.0%), the reason for adjuvant chemotherapy omission was not documented.

### Overall Survival and Recurrence in the Unadjusted Cohort

**Figure 1.**
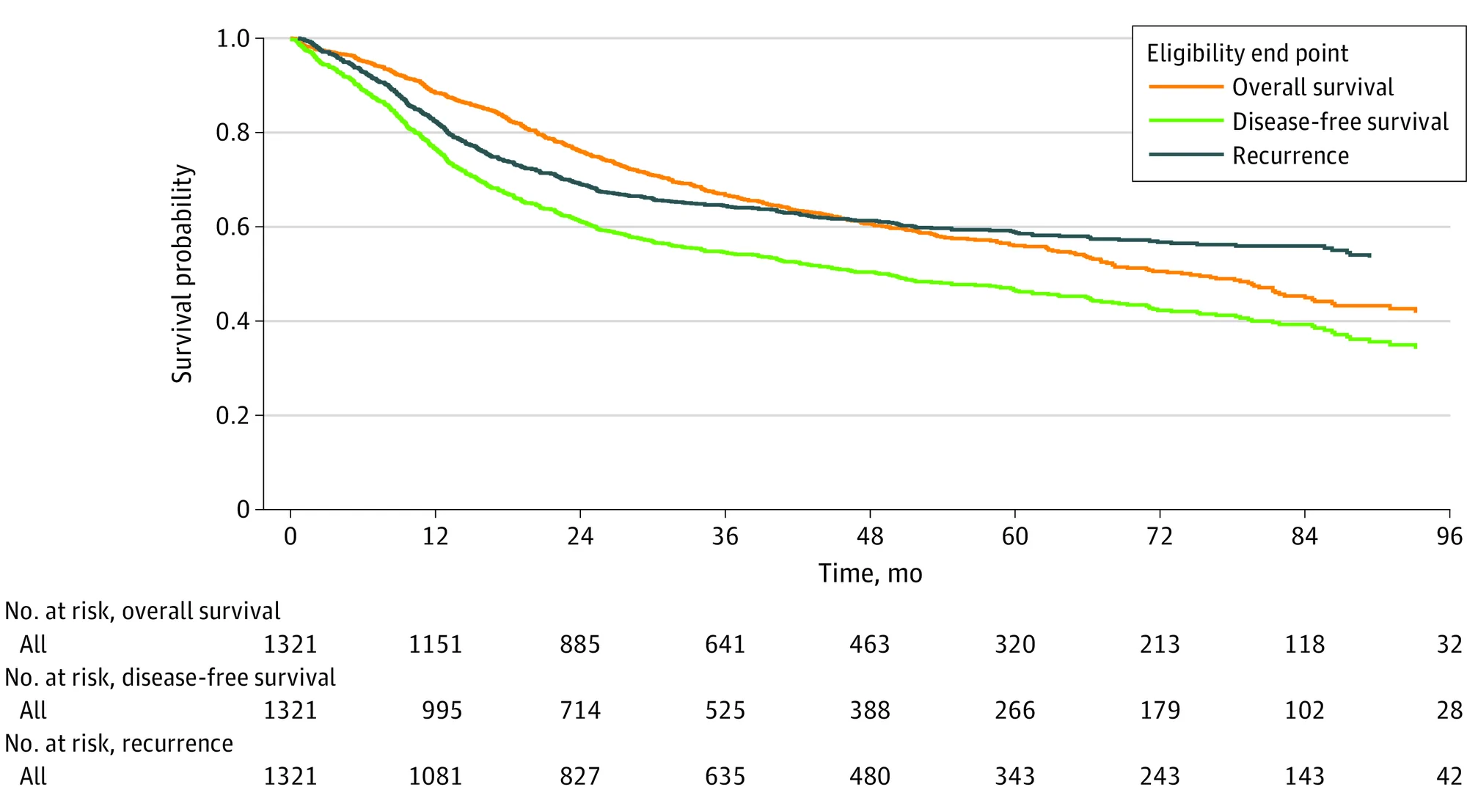
Kaplan-Meier Curves of Disease-Free Survival, Overall Survival, and Recurrence for Overall Unadjusted Cohort

The median (IQR) follow-up for the unadjusted overall cohort was 64.2 (40.4-85.0) months, and the median OS was 73.8 months (95% CI, 66.4-81.9 months) (Figure 1). Overall, 542 patients (41.0%) died during follow-up, and 1-,3-, and 5-year OS rates were 1016 of 1151 patients (88.3%), 423 of 641 patients (66.6%), and 179 of 320 patients (55.9%), respectively. There were 519 patients (39.3%) who experienced recurrence, and 1-, 3-, and 5-year recurrence rates were 192 of 1081 patients (17.8%), 227 of 635 patients (35.7%), and 142 of 343 patients (41.4%). Overall and time-specific (1-, 3-, and 5-year) recurrence rates by recurrence site are reported in eTable 2 in Supplement 1. Overall, 644 patients (48.8%) did not experience recurrence or mortality, and 1-, 3-, and 5-year DFS rates were 761 of 995 patients (76.5%), 286 of 525 patients (54.5%), and 123 of 266 patients (46.4%).

The Table highlights variables associated with mortality in univariate and multivariate analysis. In the most parsimonious multivariate model, the following variables were positively associated with mortality: tail location (hazard ratio [HR] vs other location, 1.3; 95% CI, 1.0-1.8; *P* = .049), total pancreatectomy (HR vs Whipple or PPPD, 1.5 95% CI, 1.8-1.9; *P* = .001), N1 stage (HR vs N0 stage, 1.6; 95% CI, 1.2-2.0; P < .001) or N2 (HR vs N0 stage, 2.2; 95% CI, 1.7-2.8; *P* < .001) disease, ductal type (HR vs colloid, 1.8; 95% CI, 1.2-2.5; *P* = .002), poor differentiation (HR vs other, 1.4; 95% CI, 1.1-1.7; *P*= .003), perineural invasion (HR, 2.0; 95% CI, 1.5-2.6; *P* < .001), and R1 margin (HR vs R0 margin, 1.4; 95% CI, 1.1-1.7; *P* = .004). The inclusion of these variables was supported by a DAG (eFigure 1 in Supplement 1).

To assess the potential bias from including patients ineligible for adjuvant chemotherapy due to poor fitness, 294 patients receiving adjuvant chemotherapy (all regimens) were matched with 294 patients who did not receive adjuvant chemotherapy. This was done using PSM analysis based on clinic-pathological variables (confounders) associated with OS in multivariate analysis and the DAG. In this scenario, PSM analysis demonstrated significantly longer overall survival with adjuvant chemotherapy compared with no chemotherapy (median OS, 99.7 months; 95% CI, 78.2 months to not applicable [NA] vs 51.0 months; 95% CI, 41.5 to 67.1 months; *P* = .001) (eFigure 2 in Supplement 1).

### Bias-Adjusted Cohort and Adjuvant Chemotherapy Receipt

After a 90-day landmark analysis and exclusion of patients ineligible for adjuvant chemotherapy due to poor fitness, a total of 194 patients were excluded with overlap between criteria and 1127 patients were retained for analysis. In the remaining patients, adjuvant chemotherapy receipt was more frequent in patients with higher T stage, (T3: OR vs T1, 2.41; 95% CI, 1.72-3.39; *P* < .001; T2: OR vs T1, 2.48; 95% CI, 1.81-3.40; *P* < .001), N stage 1 (OR vs N0, 2.78; 95% CI, 1.98-3.89; *P* < .001) or 2 (OR VS N0, 3.15; 95% CI, 1.89-5.26; *P* < .001) disease, moderate (OR vs well differentiated,1.86; 95% CI, 1.36-2.55; *P* < .001) or poor (OR vs well differentiated, 1.98; 95% CI, 1.37-2.87; *P* < .001) differentiation, perineural invasion (OR, 2.66; 95% CI, 2.07-3.43; *P* < .001), lymphovascular invasion (OR, 3.68; 95% CI, 2.81-4.82; *P* < .001), elevated preoperative bilirubin level (OR, 2.05; 95% CI, 1.34-3.16; *P* = .001), and R1 margin (OR, 2.09; 95% CI, 1.53-2.86; *P* < .001). Higher age (OR, 0.55; 95% CI, 0.37-0.82; *P* < .001) and higher CCI score (OR, 0.46; 95% CI, 0.30-0.71; *P* = .002) were negatively associated with receipt of adjuvant chemotherapy (eTable 3 in Supplement 1). Higher age and CCI score were defined as greater than or equal to the median compared with less than the median.

### Adjuvant Chemotherapy and Survival (Bias-Adjusted Cohort)

**Figure 2.**
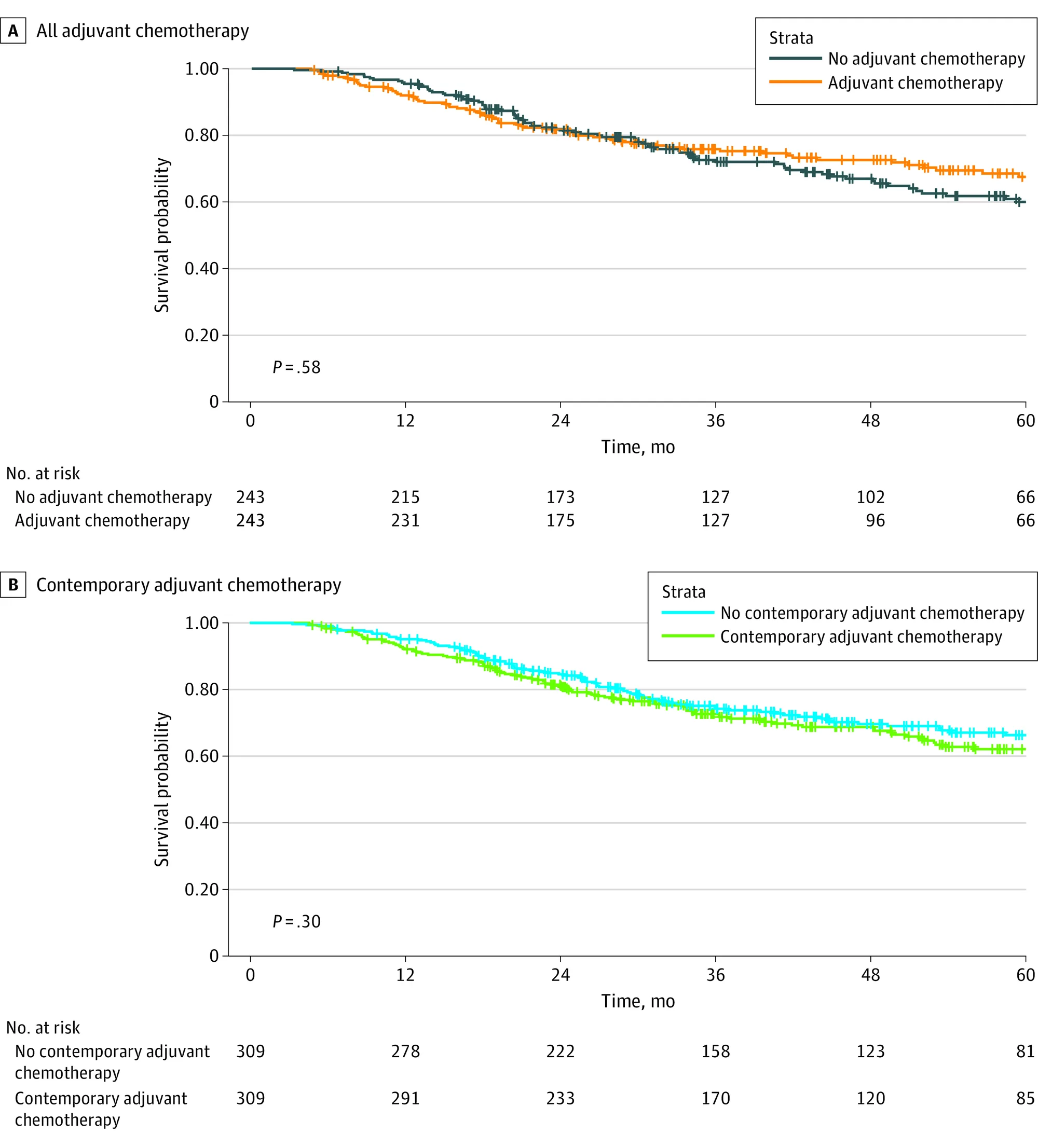
Kaplan-Meier Curves of Overall Survival in Propensity Score–Matched Analysis Curves compare all (A) and contemporary (B) adjuvant chemotherapy with no adjuvant chemotherapy.

In the bias-adjusted cohort, 243 patients receiving adjuvant chemotherapy of all regimens were matched with 243 patients who did not receive adjuvant chemotherapy using PSM analysis based on clinic-pathological variables (confounders) associated with OS. There was no difference in OS between patients receiving vs not receiving adjuvant chemotherapy (median OS, 82.3 months; 95% CI, 78.2 months to NA vs not reached; 95% CI, 75.3 months to NA; *P* = .58;) (Figure 2A). Adequate variable balancing and comparable propensity scores between groups are demonstrated in eFigure 3 in Supplement 1. Similarly, no difference in OS was noted between 309 patients receiving contemporary adjuvant chemotherapy matched with 309 patients who did not receive adjuvant chemotherapy (Figure 2B; eFigure 4 in Supplement 1). In RMST analysis, results ruled out a survival benefit associated with contemporary adjuvant chemotherapy greater than 2.1 months after 3 years and greater than 4.2 months after 5 years (difference in restricted mean survival, 1.26 months; 95%, −1.72 to 4.24 months) (eTable 4 in Supplement 1).

### Secondary Analyses

#### Subgroups by Clinicopathological Feature

Individual PSMs were performed according to clinicopathological patient subgroups following the same methodological steps. Findings were robust for age group (<60, 60-69, 70-79, and ≥80 years), sex, precursor epithelial subtype, invasive component (ductal and colloid), T stage, N stage, and margin status, and there was no association between contemporary adjuvant chemotherapy and survival in these groups (eTable 5 in Supplement 1).

Of 453 patients with node-positive disease in the bias-adjusted cohort, 371 and 82 patients received and did not receive adjuvant chemotherapy, respectively (median OS, 44.0 months; 95% CI, 37.6 to 51.6 months vs 84.6 months; 95% CI, 49.6 months to NA; *P* = .047). In PSM analyses (populations, 61:61 patients in each group), there was no difference in OS with all chemotherapy regimens compared with no adjuvant chemotherapy (median OS, 42.4 months; 95% CI, 28.7 months to NA vs 62.6 months; 95% CI, 28.7 months to NA; *P* = .46). Repeating the PSM (populations, 113:113 patients each group) but considering only contemporary chemotherapy regimens, there was also no difference observed (median OS, 46.5 months; 95% CI, 32.4 to 72.6 months vs 52.1 months; 95% CI, 32.4 months to NA; *P* = .98) (eFigure 5 in Supplement 1). This was also the case in patients who had positive lymph nodes and a high CA-19-9 level (eFigure 6 in Supplement 1).

#### Chemotherapy Regimen–Specific Analyses

**Figure 3.**
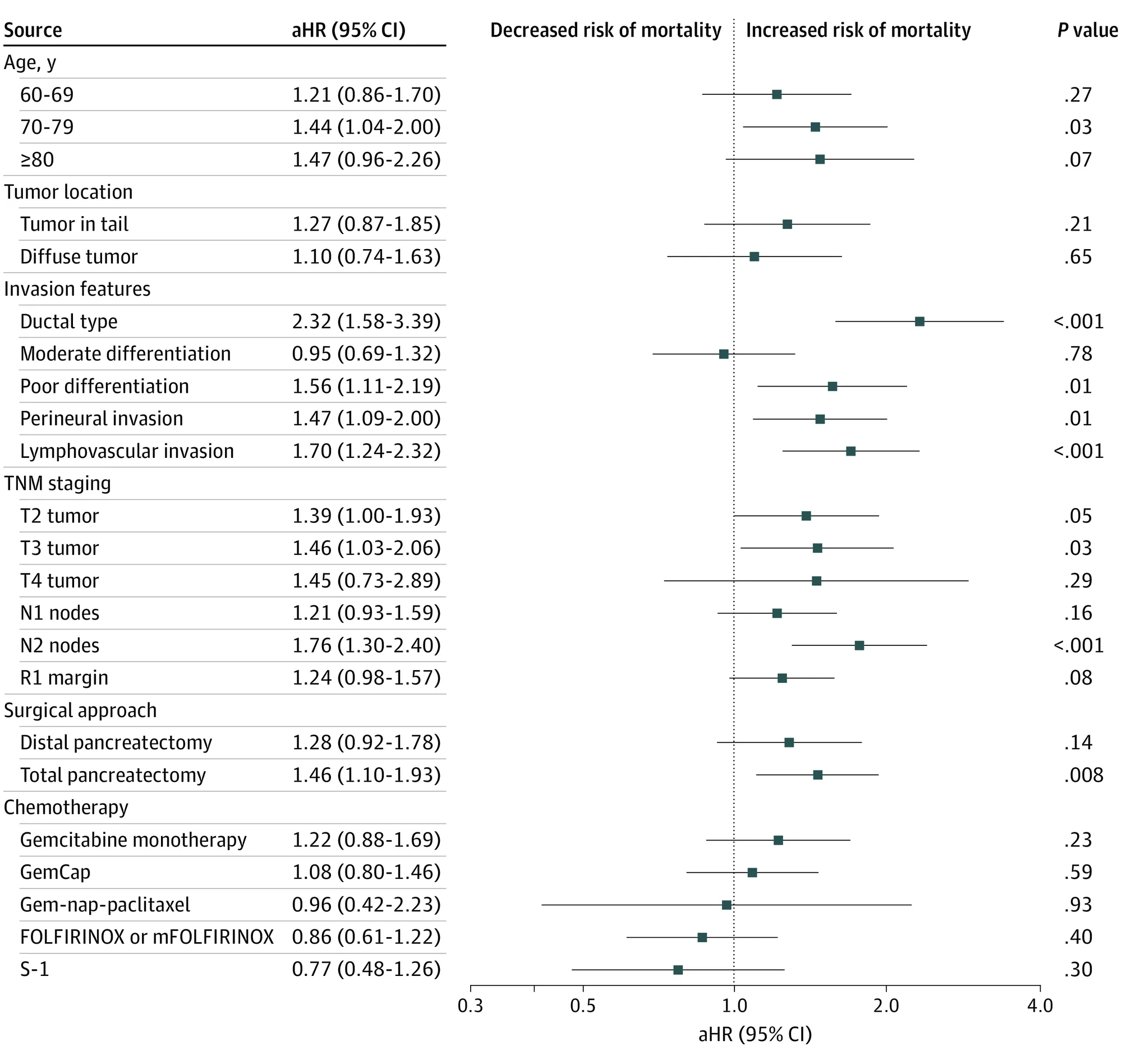
Forest Plot of Association of Variables With Mortality The plot shows a Cox proportional hazards model of variables, including adjuvant chemotherapy type, associated with mortality (performed in the bias-adjusted cohort). The reference group for all variables is no adjuvant chemotherapy. aHR indicates adjusted hazard ratio; FOLFIRINOX, 5-fluorouracil, leucovorin, oxaliplatin, and irinotecan; GemCap, gemcitabine-capecitabine; gem-nab-paclitaxel, gemcitabine with nanoparticle albumin–bound paclitaxel; mFOLFIRINOX, modified FOLFIRINOX; TNM, tumor, node, metastasis.

**Figure 4.**
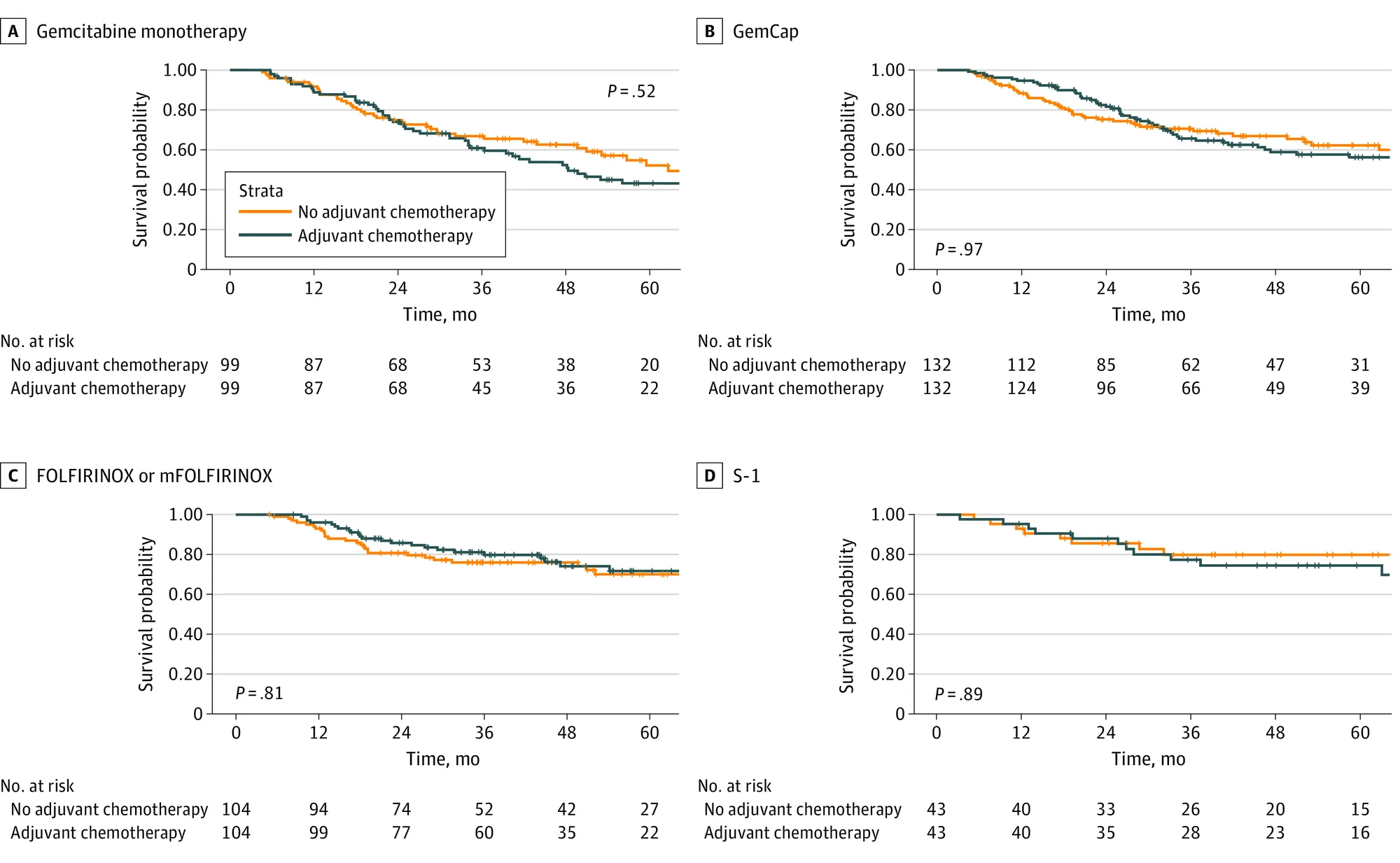
Kaplan-Meier Curves of Overall Survival by Adjuvant Chemotherapy Type After Propensity Score Matching Curves compare overall survival between adjuvant chemotherapy type and no adjuvant chemotherapy. FOLFIRINOX indicates 5-fluorouracil, leucovorin, oxaliplatin, and irinotecan; GemCap, gemcitabine-capecitabine; mFOLFIRINOX, modified FOLFIRINOX.

The median survival of each individual adjuvant chemotherapy regimen was as follows: 48.3 months (95% CI, 36.4 to 76.2 months) for gemcitabine-monotherapy, 58.0 months (95% CI, 43.1 to 81.3 months) for GemCap, 78.2 months (95% CI, 65.0 months to NA) for FOLFIRINOX or mFOLFIRINOX, and not reached for gem-nab-paclitaxel or S-1 (*P* = .003) (eFigure 7 in Supplement 1). After a Cox proportional hazards model was derived in the bias-adjusted cohort, we found that no individual adjuvant chemotherapy type was associated with a survival benefit (Figure 3). After individual PSM, no individual adjuvant chemotherapy regimen was associated with improved OS compared with no adjuvant chemotherapy (Figure 4A-D).

#### Recurrence

Recurrence was higher in the all-adjuvant chemotherapy group (median time to recurrence, 85.6 months; 95% CI, 53.1 months to NA; *P* < .001) and the contemporary adjuvant chemotherapy group (median [IQR] time to recurrence, 87.5 months; 95% CI, 53.1 months to NA; *P* = .005) compared with no adjuvant chemotherapy (median time to recurrence, not reached). This was true despite adequate PSM.

## Discussion

To our knowledge, this cohort study is the largest study to date to investigate the association of primary PDAC–derived regimens with survival outcomes after pancreatic resection for A-IPMNs. The findings do not report any improvement in OS with adjuvant chemotherapy, irrespective of adjuvant chemotherapy regimen or the presence of high-risk characteristics (eg, nodal status or CA-19-9 level). Before and after adequate PSM analysis, receipt of contemporary adjuvant chemotherapy was not associated with OS.

In conventional PDAC, adjuvant chemotherapy after resection is the standard of care.^8,9,22^ Nevertheless, evidence regarding the benefit associated with adjuvant chemotherapy in IPMN-derived PDAC is limited, and no consensus exists in current guidelines due to the paucity of high-quality studies. Despite this, PDAC-derived adjuvant chemotherapy regimens are commonly used in IPMN-derived adenocarcinoma. Administration appeared to be influenced by disease characteristics, such as higher T stage or nodal involvement (N1 or N2), while poor patient fitness remained a frequent reason for omission, in 13.7% of patients.

The major limitation of most studies investigating adjuvant chemotherapy is oversight of the fundamental selection bias for adjuvant chemotherapy. To the best of our knowledge, this is the first study to exclude patients who were deemed ineligible to receive adjuvant chemotherapy due to poor fitness.^23,24^ To demonstrate the association of not accounting for this bias with findings, a sensitivity analysis was performed including all patients who did not receive adjuvant chemotherapy regardless of their fitness. In this analysis, adjuvant chemotherapy was associated with nearly double the survival time (median OS, 99.7 vs 51.0 months; *P* = .001), even after PSM of disease characteristics. This illustrates that failing to account for treatment eligibility may generate spurious survival advantages and lead to erroneous conclusions. Therefore, extreme caution should be taken in interpreting existing literature and any future retrospective studies not excluding patients who are not candidates for adjuvant chemotherapy.

Previous studies investigating adjuvant chemotherapy for A-IPMN included data on outdated adjuvant chemotherapy regimens that are no longer preferred in patients deemed fit for adjuvant chemotherapy treatment. For example, GemCap and FOLFIRINOX constituted only 21.8% and 8.0% of regimens, respectively, in our previous publication.^14^ In a 2024 study from Habib et al,^15^ 72.4% of patients received gemcitabine monotherapy, while other studies did not report specific regimens prescribed.^16,25^ In our study, we performed a subgroup PSM analysis among most patients (568 patients [72.6%]) who received contemporary regimens as per primary PDAC trials, and no improvement in OS or recurrence was observed. In fact, recurrence rates were significantly higher in patients who received adjuvant chemotherapy. This may be explained by closer follow-up in the adjuvant chemotherapy subgroup.

Previous studies have identified an adjuvant chemotherapy benefit specifically in patients with node-positive A-IPMNs.^16,17,23,24^ A decision algorithm based on nodal status and CA-19-9 level has been published by Habib et al,^15^ whereby adjuvant chemotherapy was associated with a benefit in patients with positive lymph nodes and an increased CA-19-9 level. This finding was tested in our larger cohort of 1321 patients, and no benefit was identified for this patient subgroup.

One explanation for the poor response to adjuvant chemotherapy could be the heterogenous nature of A-IPMN. A-IPMNs have different immunohistochemical and morphological characteristics that are associated with the final oncological and survival outcome. For example, the pancreatobiliary subtype is associated with poorer survival, while the intestinal subtype may be associated with a favorable prognosis compared with the gastric subtype.^3,26,27^ One hypothesis is that adjuvant chemotherapy response varies by precursor epithelial subtype or the invasive component. Our previous publication indicated that the pancreatobiliary subtype may have a superior response to adjuvant chemotherapy due to their similar molecular phenotypes and mutational status to primary PDAC and ampullary adenocarcinoma.^28,29^ However, subgroup analysis of more than 500 patients with the pancreatobiliary subtype in this study did not support this association.

In existing guidelines on pancreatic cysts management, the Fukuoka consensus statement and the American College of Gastroenterology clinical guidelines do not make recommendations on the use of adjuvant chemotherapy in A-IPMN. The 2024 updated Kyoto guidelines reported that the role of adjuvant chemotherapy in resectable A-IPMN is unknown.^13^ However, European guidelines recommend adjuvant chemotherapy with PDAC regimens for A-IPMNs regardless of nodal status.^10,11,12^ In light of the findings of our large cohort study, reevaluation of adjuvant chemotherapy guidelines for A-IPMNs may be warranted. Randomized clinical trials may be required to investigate the role of new chemotherapy agents rather than PDAC-derived adjuvant chemotherapy regimens in IPMN-derived adenocarcinoma.

### Limitations

This study has several limitations. Selection bias was addressed in this study by excluding patients deemed unfit for adjuvant chemotherapy and by performing a landmark analysis to mitigate immortal time bias. Nonetheless, as with all retrospective observational studies, residual selection bias is likely, including unmeasured confounding from factors not captured in the dataset (eg, performance status differences, center-specific treatment policies, and patient selection). For example, variable timing of chemotherapy initiation and the different surveillance protocols after chemotherapy were not captured. Other limitations include potential misclassification bias arising from variability in pathological assessment and the absence of central radiological or histological review. Furthermore, although contemporary regimens were analyzed separately, changes in surgical techniques and decision-making between centers could have influenced outcomes independently of chemotherapy use. Additionally, S-1 chemotherapy is specific to certain geographic regions, such as Japan, and the association of S-1 with outcomes may not be fully comparable to that of other contemporary chemotherapy regimens.

## Conclusions

The findings of this cohort study call into question the use of PDAC-derived adjuvant chemotherapy regimens in A-IPMNs. Despite adequate PSM analysis, an analysis of predominantly contemporary adjuvant chemotherapy regimens, and the adjustment for selection bias for adjuvant chemotherapy receipt, we found that adjuvant chemotherapy was not associated with improved survival. High-risk groups that have been purported to benefit from adjuvant chemotherapy did not have a higher survival with adjuvant chemotherapy in this study. These findings support the need for a future randomized clinical trial to investigate the impact of adjuvant chemotherapy in A-IPMNs.

## Notes

Supplement 1. **eTable 1.** Missing data for variables included in PSM **eTable 2.** Overall and time-specific (1-, 3- and 5-year) recurrence rate by recurrence site **eTable 3.** Variables associated with administration of adjuvant chemotherapy in the bias-adjusted cohort **eTable 4.** Difference in restricted mean survival time by follow-up for all adjuvant chemotherapy and contemporary adjuvant chemotherapy regimens **eTable 5.** Log-rank and RMST analyses performed in patient subgroups after individual PSMs performed for each subgroup **eFigure 1.** Directed acyclic graph including exposure (chemotherapy), outcome (OS), confounders, and redundant covariates **eFigure 2.** Overall survival in PSM analysis comparing all (A) and contemporary (B) adjuvant chemotherapy with no adjuvant chemotherapy in the unadjusted cohort before exclusions **eFigure 3.** Jitter (A) and Love (B) plot of propensity scores in PSM models of adjuvant chemotherapy (any or all adjuvant chemotherapy) vs no adjuvant chemotherapy (conducted in the bias-adjusted cohort). Variable balancing (C) between treated (any or all adjuvant chemotherapy) and control (no adjuvant chemotherapy) groups **eFigure 4.** Jitter (A) and Love (B) plot of propensity scores in PSM models of adjuvant chemotherapy (contemporary) vs no adjuvant chemotherapy (conducted in the bias-adjusted cohort). Variable balancing (C) between treated (contemporary adjuvant chemotherapy) and control (no adjuvant chemotherapy) groups **eFigure 5.** Comparison of overall survival in patients with node-positive disease between all (A) and contemporary (B) adjuvant chemotherapy vs no chemotherapy (conducted in the bias-adjusted cohort). PSM comparison in patients with node-positive disease between all (C) and contemporary (D) adjuvant chemotherapy regimens vs no chemotherapy **eFigure 6.** Kaplan-Meier curve of patients with node-positive disease with high CA-19-9 levels (≥37 U/ml) with and without adjuvant chemotherapy **eFigure 7.** Comparison of overall survival between chemotherapy regimens

Supplement 2. **Nonauthor Collaborators.** Adeno-IPMN2 Collaborative

Supplement 3.
Data Sharing Statement
