# Error in Supplement 2

**DOI:** 10.1001/jamanetworkopen.2026.44280

**Published:** 2026-10-19

In the Original Investigation titled “Contemporary Adjuvant Chemotherapy for Intraductal Papillary Mucinous Neoplasms,”^1^ published March 27, 2026, nonauthor collaborator Ahmet Bulent Dogrul’s name was misspelled in Supplement 2. The article has been corrected.^1^
