# Supplementary material for: Contemporary Adjuvant Chemotherapy for Intraductal Papillary Mucinous Neoplasms
Source: JAMA Netw Open. 2026 Mar 27;9(3):e263688. doi: 10.1001/jamanetworkopen.2026.3688 (PMC13032148; doi:10.1001/jamanetworkopen.2026.3688)
Supplement: Supplement 1. — eTable 1. Missing data for variables included in PSM eTable 2. Overall and time-specific (1-, 3- and 5-year) recurrence rate by recurrence site eTable 3. Variables associated with administration of adjuvant chemotherapy in the bias-adjusted cohort eTable 4. Difference in restricted mean survival time by follow-up for all adjuvant chemotherapy and contemporary adjuvant chemotherapy regimens eTable 5. Log-rank and RMST analyses performed in patient subgroups after individual PSMs performed for each subgroup eFigure 1. Directed acyclic graph including exposure (chemotherapy), outcome (OS), confounders, and redundant covariates eFigure 2. Overall survival in PSM analysis comparing all (A) and contemporary (B) adjuvant chemotherapy with no adjuvant chemotherapy in the unadjusted cohort before exclusions eFigure 3. Jitter (A) and Love (B) plot of propensity scores in PSM models of adjuvant chemotherapy (any or all adjuvant chemotherapy) vs no adjuvant chemotherapy (conducted in the bias-adjusted cohort). Variable balancing (C) between treated (any or all adjuvant chemotherapy) and control (no adjuvant chemotherapy) groups eFigure 4. Jitter (A) and Love (B) plot of propensity scores in PSM models of adjuvant chemotherapy (contemporary) vs no adjuvant chemotherapy (conducted in the bias-adjusted cohort). Variable balancing (C) between treated (contemporary adjuvant chemotherapy) and control (no adjuvant chemotherapy) groups eFigure 5. Comparison of overall survival in patients with node-positive disease between all (A) and contemporary (B) adjuvant chemotherapy vs no chemotherapy (conducted in the bias-adjusted cohort). PSM comparison in patients with node-positive disease between all (C) and contemporary (D) adjuvant chemotherapy regimens vs no chemotherapy eFigure 6. Kaplan-Meier curve of patients with node-positive disease with high CA-19-9 levels (≥37 U/ml) with and without adjuvant chemotherapy eFigure 7. Comparison of overall survival between chemotherapy regimens [file jamanetwopen-e263688-s001.pdf]

Supplemental Online Content

Lucocq J, Haugk B, White S, et al. Contemporary adjuvant chemotherapy for intraductal papillary mucinous neoplasms. *JAMA Netw Open*. 2026;9(3):e263688. doi:10.1001/jamanetworkopen.2026.3688

eTable 1. Missing data for variables included in PSM

eTable 2. Overall and time-specific (1-, 3- and 5-year) recurrence rate by recurrence site

eTable 3. Variables associated with administration of adjuvant chemotherapy in the bias-adjusted cohort

eTable 4. Difference in restricted mean survival time by follow-up for all adjuvant chemotherapy and contemporary adjuvant chemotherapy regimens

eTable 5. Logrank and RMST analyses performed in patient subgroups after individual PSMs performed for each subgroup

eFigure 1. Directed acyclic graph including exposure (chemotherapy), outcome (OS), confounders, and redundant covariates

eFigure 2. Overall survival in PSM analysis comparing all (A) and contemporary (B) adjuvant chemotherapy with no adjuvant chemotherapy in the unadjusted cohort before exclusions

eFigure 3. Jitter (A) and Love (B) plot of propensity scores in PSM models of adjuvant chemotherapy (any or all adjuvant chemotherapy) vs no adjuvant chemotherapy (conducted in the bias-adjusted cohort). Variable balancing (C) between treated (any or all adjuvant chemotherapy) and control (no adjuvant chemotherapy) groups

eFigure 4 . Jitter (A) and Love (B) plot of propensity scores in PSM models of adjuvant chemotherapy (contemporary) vs no adjuvant chemotherapy (conducted in the bias-adjusted cohort). Variable balancing (C) between treated (contemporary adjuvant chemotherapy) and control (no adjuvant chemotherapy) groups

eFigure 5. Comparison of overall survival in patients with node-positive disease between all (A) and contemporary (B) adjuvant chemotherapy vs no chemotherapy (conducted in the bias-adjusted cohort). PSM comparison in patients with node-positive disease between all (C) and contemporary (D) adjuvant chemotherapy regimens vs no chemotherapy

eFigure 6. Kaplan-Meier curve of patients with node-positive disease with high CA-19-9 levels (≥37 U/ml) with and without adjuvant chemotherapy

eFigure 7. Comparison of overall survival between chemotherapy regimens

This supplemental material has been provided by the authors to give readers additional information about their work.

© 2026 Lucocq J et al. *JAMA Network Open*.

Supplementary Table 1. Missing data for variables included in PSM

Variable	Number (%) (N = 1321)
**Median age, Q1-3**	0 (0.0)
**Sex**	0 (0.0)
**Tumour location (e.g. head, body, tail)**	3 (0.2)
**Operation**	3 (0.2)
**Differentiation**	53 (4.0)
**Invasive component**	103 (7.8)
**T stage**	17 (1.3)
**N stage**	5 (0.4)
**Perineural invasion**	21 (1.6)
**Lymphovascular invasion**	22 (1.7)
**R1**	5 (0.4)

© 2026 Lucocq J et al. *JAMA Network Open*.

Supplementary Table 2: Overall and time-specific (1-, 3- and 5-year) recurrence rate by recurrence site

Recurrence	Overall Number (%)	Time-specific recurrence rate		
		1 year	3 year	5 year
**Local recurrence**	186 (14.8)	6.6%	14.5%	16.9%
· **Remnant pancreas**	71 (5.4)	2.4%	5.5%	7.0%
· **Pancreatic bed**	93 (7.0)	3.1%	8.1%	9.0%
· **Peripancreatic lymph nodes**	30 (2.3)	1.5%	2.4%	2.5%
**Distant recurrence**	389 (29.4)	14.0%	28.4%	32.6%
· **Liver**	183 (13.9)	7.8%	15.2%	16.1%
· **Lung**	117 (8.9)	3.7%	8.7%	11.4%
· **Peritoneal**	85 (6.4)	3.4%	7.3%	8.3%
· **Extrapancreatic lymph nodes**	39 (3.0)	1.4%	3.1%	4.0%
· **Other site**	37 (2.8)	1.3%	3.2%	4.1%

© 2026 Lucocq J et al. *JAMA Network Open*.

Supplementary Table 3. Variables associated with administration of adjuvant chemotherapy in the bias-adjusted cohort

Variable		OD	Std. Error	z-value	P-value
**Median age, Q1-3**		0.55	0.20	-3.52	<0.001
**Median CCI**		0.46	0.21	-3.78	0.002
**Location**	Head	Reference			
	Body	1.06	0.18	0.31	0.76
	Tail	1.05	0.20	0.23	0.82
	Diffuse	1.15	0.26	0.56	0.58
**Duct type**	Main	Reference			
	Branch	1.12	1.19	0.63	0.53
	Mixed	1.12	1.14	0.85	0.40
**Operation**	Whipples/PPPD	Reference			
	DP	1.19	0.16	1.13	0.26
	TP	0.79	0.17	-1.38	0.17
**Bilirubin elevated***		2.05	0.22	3.27	0.001
**Ca 19-9**	37-200	1.03	1.17	0.17	0.87
	>200	1.36	1.18	1.87	0.06
**T stage**	T1	Reference			
	T2	2.48	0.16	5.87	<0.001
	T3	2.41	0.17	5.20	<0.001
	T4	2.34	0.59	1.45	0.15
**N stage**	N0	Reference			
	N1	2.78	0.17	6.10	<0.001
	N2	3.15	0.26	5.09	<0.001
**Precursor subtype**	Gastric	Reference			
	Intestinal	0.75	0.22	-1.31	0.19
	Pancreatobiliary	0.92	0.18	-0.47	0.64
	Mixed	1.01	0.29	-0.05	0.956
**Invasive component**	Colloid	Reference	-	-	-
	Ductal	1.60	0.16	2.90	0.004
**Differentiation**	Well	Reference			
	Moderate	1.86	0.16	3.81	<0.001
	Poor	1.98	0.19	3.54	<0.001
**Perineural invasion**		2.66	0.13	7.33	<0.001
**Lymphovascular invasion**		3.68	0.14	9.22	<0.001
**R1 Margin**		2.09	0.16	4.70	<0.001

© 2026 Lucocq J et al. *JAMA Network Open*.

Supplementary Table 4: Difference in restricted mean survival time by follow-up for all adjuvant chemotherapy and contemporary adjuvant chemotherapy regimens.

**Adjuvant Chemotherapy Regimens Included**	**Follow-up horizon (months)**	**RMST difference (months)**	**95% CI (months)**	**p-value**	**Maximum survival benefit excluded (months)**
All Adjuvant Chemotherapy	12	0.15	−0.04 to 0.34	0.13	>0.34
	24	0.46	−0.31 to 1.23	0.24	>1.23
	36	0.42	−1.14 to 1.98	0.60	>1.98
	48	−0.06	−2.51 to 2.39	0.96	>2.39
	60	−0.94	−4.34 to 2.46	0.59	>2.46
Contemporary Adjuvant Chemotherapy	12	0.12	−0.06 to 0.29	0.19	>0.29
	24	0.50	−0.18 to 1.17	0.15	>1.17
	36	0.74	−0.61 to 2.09	0.28	>2.09
	48	0.91	−1.22 to 3.05	0.40	>3.05
	60	1.26	−1.72 to 4.24	0.41	>4.24

© 2026 Lucocq J et al. *JAMA Network Open*.

Supplementary Table 5: Logrank and RMST analyses performed in patient subgroups following individual PSMs performed for each subgroup.

Subgroup		Treated, n	Control, n	Logrank p value	RMST at 60 months		
					Difference in mean survival	95%CI	p-value
Sex	Female	105	105	0.92	0.61	-4.41 to 5.62	0.81
	Male	120	120	0.1	2.88	-1.76 to 7.53	0.22
Age	<60	19	19	0.92	3.9	-5.32 to 13.11	0.41
	60-69	52	52	0.69	-0.31	-6.75 to 6.13	0.92
	70-79	109	109	0.78	-0.24	-5.81 to 5.33	0.93
	>=80	21	21	0.91	-1.41	-13.29 to 10.48	0.82
T stage	T1	88	88	0.38	0.94	-3.46 to 5.34	0.67
	T2	72	72	0.57	2.03	-4.87 to 8.93	0.56
	T3	40	40	0.5	-2.92	-12.27 to 6.44	0.54
N stage	N0	127	127	0.17	1.38	-2.58 to 5.34	0.5
	Node-positive	113	113	0.23	-5.07	-12.33 to 2.19	0.17
Histology	Colloid	41	41	0.85	-0.14	-5.41 to 5.13	0.96
	Ductal	176	176	0.89	-0.19	-4.43 to 4.06	0.93
Pancreatobiliary subtype		77	77	0.82	-0.69	-7.23 to 5.86	0.84
R status	R0	177	177	0.4	1.15	-2.62 to 4.92	0.55
	R1	39	39	0.4	0.39	-9.09 to 9.87	0.94

© 2026 Lucocq J et al. *JAMA Network Open*.

Supplementary Figure 1. Directed Acyclic Graph including Exposure (Chemotherapy), Outcome (OS), Confounders and Redundant Co-variates.

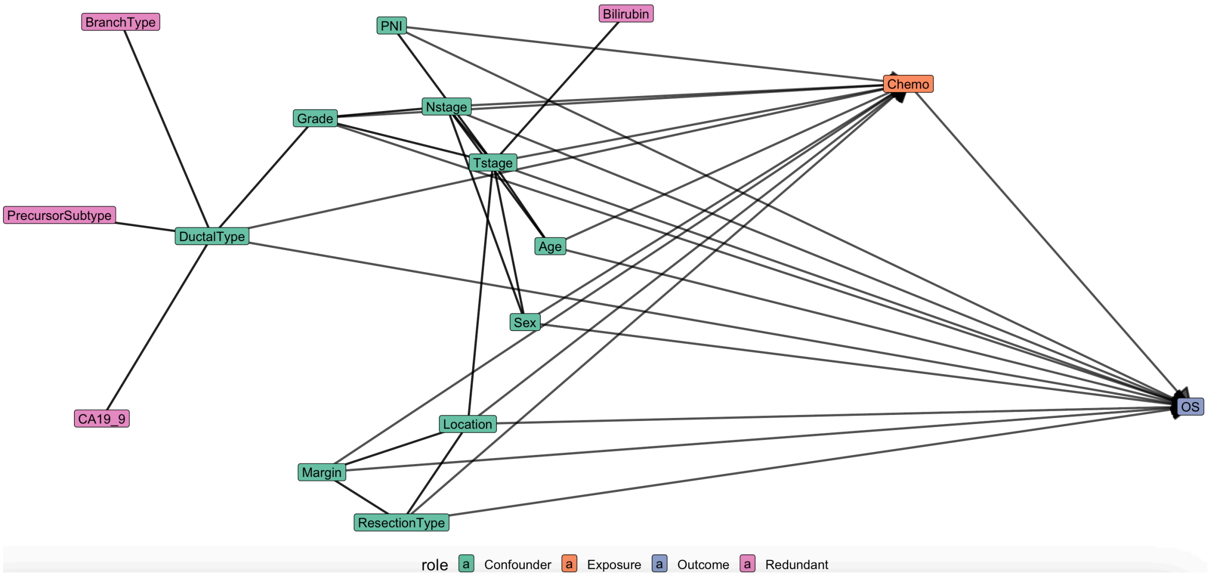

© 2026 Lucocq J et al. *JAMA Network Open*.

Supplementary Figure 2. Overall survival in PSM analysis comparing all (A) and contemporary (B) adjuvant chemotherapy with no adjuvant chemotherapy in the unadjusted cohort before exclusions

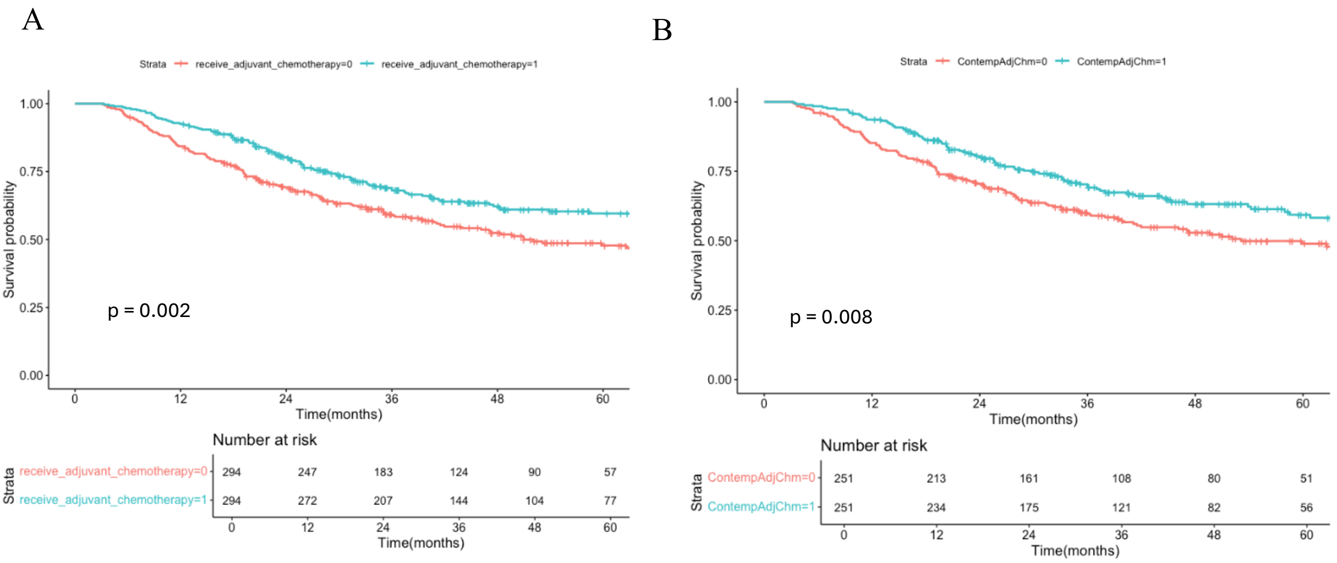

© 2026 Lucocq J et al. *JAMA Network Open*.

Supplementary Figure 3. Jitter (A) and Love (B) Plot of Propensity Scores in PSM models of adjuvant chemotherapy (any or all adjuvant chemotherapy) versus no adjuvant chemotherapy (conducted in the bias-adjusted cohort). Variable balancing (C) between treated (any or all adjuvant chemotherapy) and control (no adjuvant chemotherapy) groups.

A

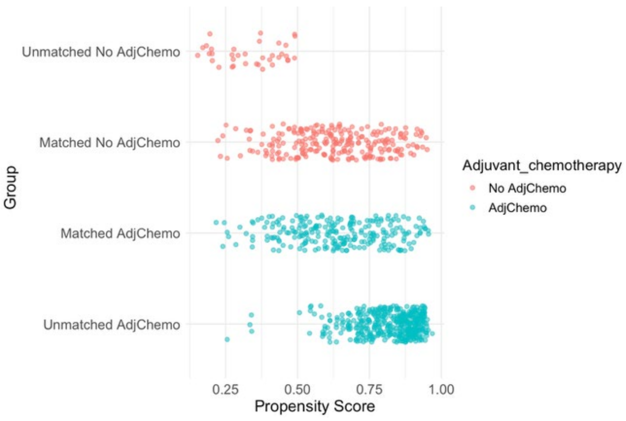

B

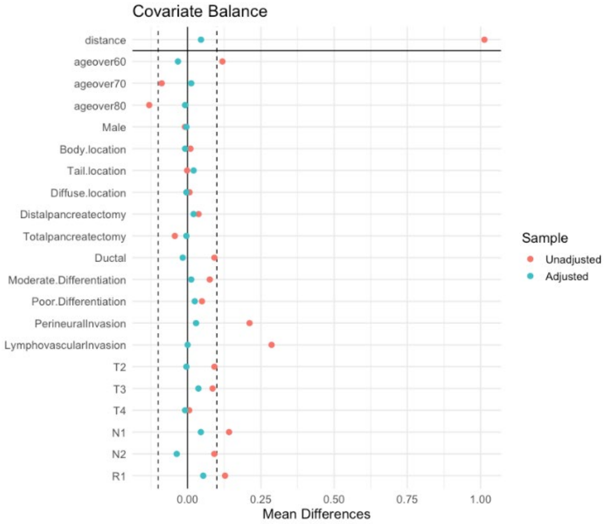

C

Variable		Means Treated	Means Control	Std. Mean Diff.	eCDF Mean	eCDF Max
**Age**	60–69	54 (22.2)	62 (25.5)	-0.07	0.03	0.03
	70–79	120 (49.4)	117 (48.1)	0.03	0.01	0.01
	≥80	33 (13.6)	35 (14.4)	-0.03	0.01	0.01
**Male**		129 (53.1)	130 (53.5)	-0.01	0.00	0.00
**Location**	Body	36 (14.8)	38 (15.6)	-0.02	0.01	0.01
	Tail	38 (15.6)	33 (13.6)	0.06	0.02	0.02
	Diffuse	16 (6.6)	17 (7.0)	-0.02	0.00	0.00
**Operation**	Distal pancreatectomy	62 (25.5)	57 (23.5)	0.05	0.02	0.02
	Total pancreatectomy	50 (20.6)	51 (21.0)	-0.01	0.01	0.00
**Invasive Characteristics**	Ductal type	180 (74.1)	184 (75.7)	-0.04	0.02	0.02
	Moderate differentiation	118 (48.6)	115 (47.3)	0.03	0.01	0.01
	Poor differentiation	57 (23.5)	51 (21.0)	0.06	0.03	0.03
	Perineural invasion	124 (51.0)	117 (48.1)	0.06	0.03	0.03
**Stage**	T2	82 (33.7)	83 (34.2)	-0.08	0.00	0.00
	T3	61 (25.1)	52 (21.4)	0.08	0.04	0.04
	T4	1 (0.4)	3 (1.2)	-0.07	0.01	0.01
	N1	55 (22.6)	44 (18.1)	0.10	0.05	0.05
	N2	16 (6.6)	25 (10.3)	-0.10	0.04	0.04
**R1 margin**		65 (26.7)	52 (21.4)	0.11	0.05	0.05

© 2026 Lucocq J et al. *JAMA Network Open*.

Supplementary Figure 4. Jitter (A) and Love (B) Plot of Propensity Scores in PSM models of adjuvant chemotherapy (contemporary) versus no adjuvant chemotherapy (conducted in the bias-adjusted cohort). Variable balancing (C) between treated (contemporary adjuvant chemotherapy) and control (no adjuvant chemotherapy) groups.

A

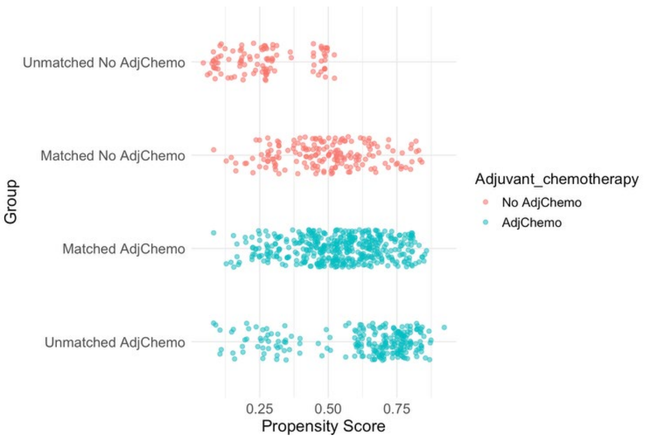

B

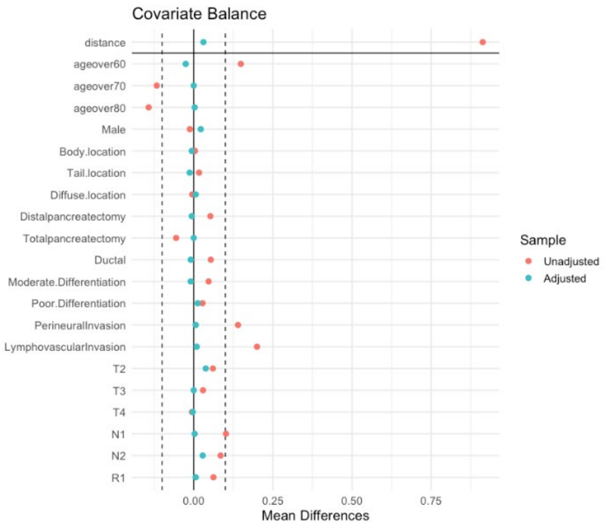

C

Variable		Means Treated (%)	Means Control (%)	Std. Mean Diff.	eCDF Mean	eCDF Max
**Age**	60-69	85 (27.5)	93 (30.1)	0.03	0.03	0.69
	70-79	150 (48.4)	150 (48.4)	0.00	0.00	0.35
	≥80	18 (5.7)	17 (5.4)	0.00	0.00	0.27
**Male**		172 (55.7)	165 (53.5)	0.02	0.02	0.86
**Location**	Body	47 (15.2)	49 (15.8)	0.01	0.01	0.66
	Tail	39 (12.7)	43 (13.9)	0.01	0.01	0.66
	Diffuse	22 (7.0)	19 (6.3)	0.01	0.01	0.43
**Operation**	Distal pancreatectomy	76 (24.7)	78 (25.3)	0.01	0.01	0.70
	Total pancreatectomy	60 (19.3)	60 (19.3)	0.00	0.00	0.32
**Invasive characteristics**	Ductal type	239 (77.5)	243 (78.5)	0.01	0.01	0.84
	Moderate Differentiation	155 (50.0)	157 (50.9)	0.01	0.01	0.94
	Poor differentiation	74 (24.1)	70 (22.8)	0.01	0.01	0.76
	Perineural Invasion	173 (56.0)	171 (55.4)	0.01	0.01	0.87
**Stage**	T2	120 (38.9)	108 (35.1)	0.04	0.04	0.88
	T3	83 (26.9)	83 (26.9)	0.00	0.00	0.37
	T4	3 (0.9)	4 (1.3)	0.00	0.00	0.20
	N1	79 (25.6)	78 (25.3)	0.00	0.00	0.70
	N2	52 (16.8)	43 (13.9)	0.03	0.03	0.60
**R1 margin**		89 (28.8)	87 (28.2)	0.01	0.01	0.80

© 2026 Lucocq J et al. *JAMA Network Open*.

Supplementary Figure 5. Comparison of overall survival in node-positive patients, between all (A) and contemporary (B) adjuvant chemotherapy versus no chemotherapy (conducted in the bias-adjusted cohort). PSM comparison in node-positive patients between all (C) and contemporary (D) adjuvant chemotherapy regimens versus no chemotherapy.

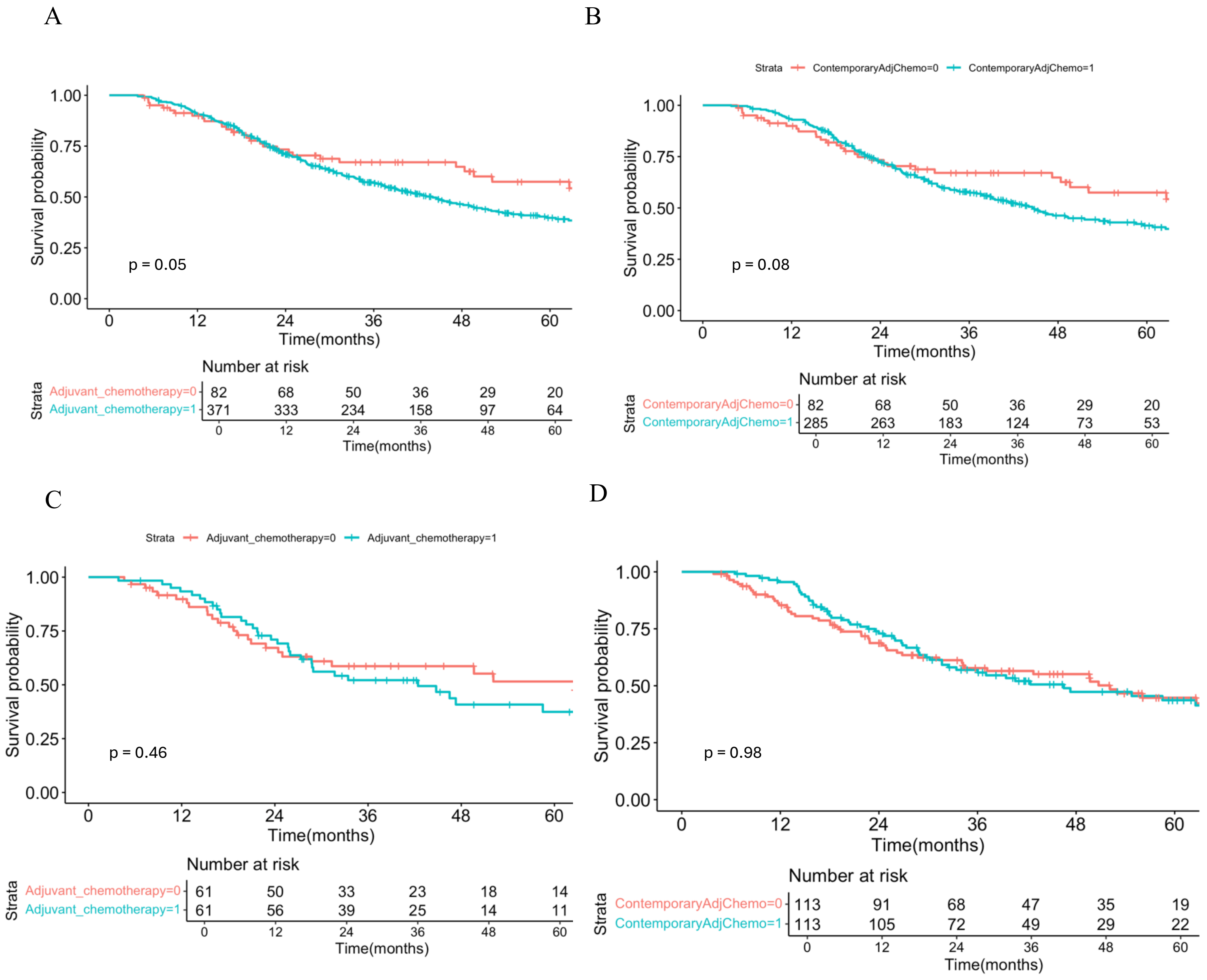

© 2026 Lucocq J et al. *JAMA Network Open*.

Supplementary Figure 6. Kaplan-Meier curve of node-positive patients with high CA-19-9 (≥37U/ml) with and without adjuvant chemotherapy

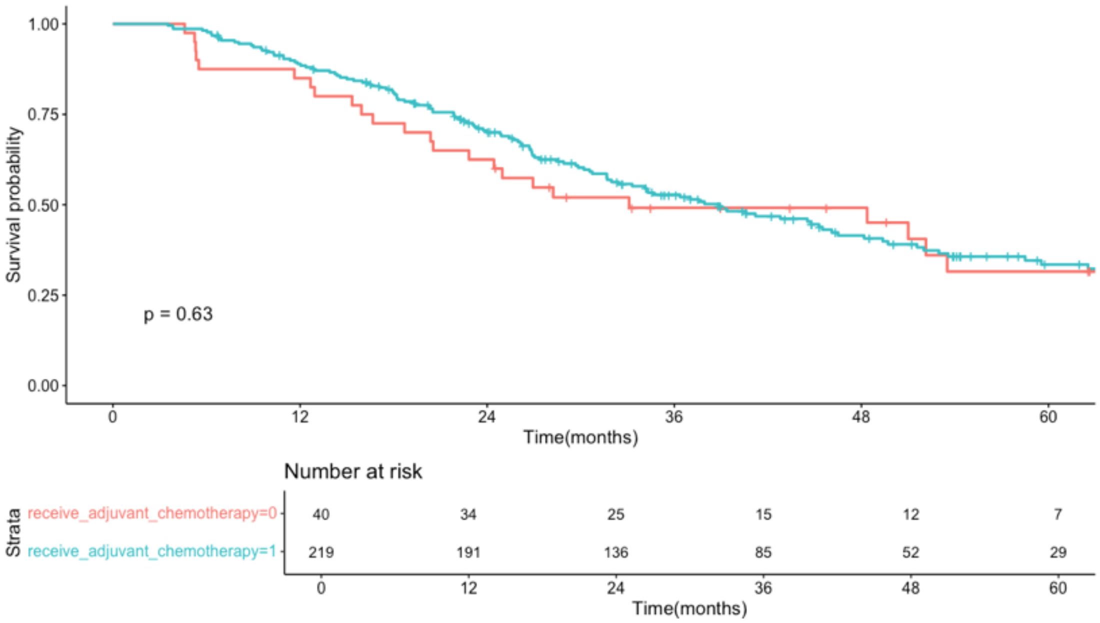

© 2026 Lucocq J et al. *JAMA Network Open*.

Supplementary Figure 7. Comparison of overall survival between chemotherapy regimens

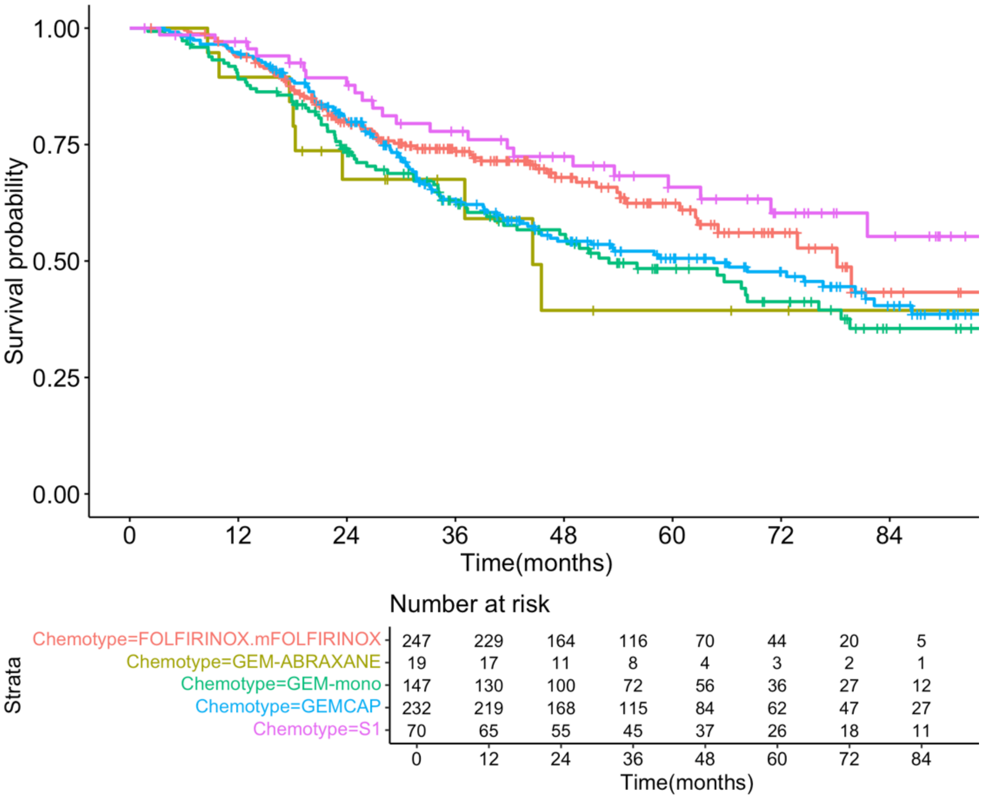

© 2026 Lucocq J et al. *JAMA Network Open*.
