# Supplementary material for: Contemporary Adjuvant Chemotherapy for Intraductal Papillary Mucinous Neoplasms
Source: JAMA Netw Open. 2026 Mar 27;9(3):e263688. doi: 10.1001/jamanetworkopen.2026.3688 (PMC13032148; doi:10.1001/jamanetworkopen.2026.3688)
Supplement: Supplement 2. — Nonauthor Collaborators. Adeno-IPMN2 Collaborative [file jamanetwopen-e263688-s002.pdf]

*Group Name(s): Adeno-IPMN2 Collaborative							
***First Name and Middle Initial(s)**	***Last Name**	***Suffix (eg, Jr, III)**	Academic Degrees	Institution	Location (city, state/province, country)	Role or Contribution, eg, chair, principal investigator	Group (if more than 1 Group listed in the byline) and/or Subgroup (eg, Steering Committee)
Ajit	Kumar			All India Institute of Medical Sciences Rishikesh, India			
Hüseyin	Fahri Martlı			Ankara Bilkent City Hospital, Türkiye			
Geert	Roeyen			Antwerp University Hospital, Edegem, Belgium			
Pantelis	Antonakis			National & Kapodistrian University of Athens, Aretaieion Hospital, Greece			
Konstantinos	Bramis			National & Kapodistrian University of Athens, Aretaieion Hospital, Greece			
Carol	González			HPB and Transplant Surgery, Hospital Clinic Barcelona, IDIBAPS, University of Barcelona, Spain			
James	Halle-Smith			University Hospitals Birmingham, UK			
Shi	Lam			Cambridge HPB Unit, Cambridge University Hospitals NHS Foundation Trust, Cambridge, United Kingdom			
Ivan	L'Official-German			Capital Health Cancer Center, Pennington, NJ, USA			
Tina	Bergner			Capital Health Cancer Center, Pennington, NJ, USA			
Edoardo	Rosso			Centre de Chirurgie Digestive, Le Mans, France			

***First Name and Middle Initial(s)**	***Last Name**	***Suffix (eg, Jr, III)**	Academic Degrees	Institution	Location (city, state/province, country)	Role or Contribution, eg, chair, principal investigator	Group (if more than 1 Group listed in the byline) and/or Subgroup (eg, Steering Committee)
Valentina	Valle			Division of General Surgery, Minimally Invasive and Robotic Surgery, University of Illinois, Chicago, USA			
Pier Cristoforo	Giulianotti			Division of General Surgery, Minimally Invasive and Robotic Surgery, University of Illinois, Chicago, USA			
Mathew	Morreau			Christchurch Hospital, New Zealand			
Saxon	Connor			Christchurch Hospital, New Zealand			
Alexandra	Dili			CHU UCL Namur - Godinne, Belgium			
Maria	Sotiropoulou			Evaggelismos Hospital, Athens, Greece			
Stylianos	Kapiris			Evaggelismos Hospital, Athens, Greece			
Matta	Kuzman			Freeman Hospital, UK			
Ahmet Çağrı	Büyükkasap			Gazi University, Ankara, Turkey			
Gökhan	Uludağ			Gazi University, Ankara, Turkey			
Mustafa	Kerem			Gazi University, Ankara, Turkey			
Mari-Claire	McGuigan			Glasgow Royal Infirmary, UK			
Tarak	Chouari			Hepato-Pancreato-Biliary (HPB) Surgical Unit, Royal Surrey NHS Foundation Trust, Guildford, UK			
Ahmet	Dogul			Hacettepe University Department of General Surgery, Ankara, Türkiye			

***First Name and Middle Initial(s)**	***Last Name**	***Suffix (eg, Jr, III)**	Academic Degrees	Institution	Location (city, state/province, country)	Role or Contribution, eg, chair, principal investigator	Group (if more than 1 Group listed in the byline) and/or Subgroup (eg, Steering Committee)
Dogukan	Dogu			Hacettepe University Department of General Surgery, Ankara, Türkiye			
Artur	Rebelo			Department of Visceral, Vascular and Endocrine Surgery, University Hospital Halle (Saale), Martin-Luther-University Halle-Wittenberg, Germany			
Franziska Bernadette	Holewik			Department of Visceral, Vascular and Endocrine Surgery, University Hospital Halle (Saale), Martin-Luther-University Halle-Wittenberg, Germany			
Roxana	Plesa-Furda			Hautepierre Hospital, Strasbourg, France			
Omero Pereira da Costa	Filho			Military Hospital of Porto Alegre (HMAPA), Brazil			
Fernando	Revoredo			Hospital Nacional Guillermo Almenara Irigoyen, Lima, Peru			
Ilias	Galanis			Hygeia Hospital, Athens, Greece			
Dhiresh	Maharjan			Department of General and HPB Surgery, Kathmandu, Nepal			
Poya	Ghorbani			Department of HPB Surgery, Karolinska Institute, Sweden			
Max	Lind			Department of HPB Surgery, Karolinska Institute, Sweden			

***First Name and Middle Initial(s)**	***Last Name**	***Suffix (eg, Jr, III)**	Academic Degrees	Institution	Location (city, state/province, country)	Role or Contribution, eg, chair, principal investigator	Group (if more than 1 Group listed in the byline) and/or Subgroup (eg, Steering Committee)
Katsuya	Ami			Kindai University Faculty of Medicine, Osaka, Japan			
Aarathi	Vijayashanker			Kings College Hospital, London, UK			
Tareq	Alsaoudi			Leicester General Hospital, Leicester, UK			
Linda	Lundgren			Department of Surgery and Clinical and Experimental Medicine, Linköping University, Linköping, Sweden			
Katrina	Ellisa			Makati Medical Center, Philippines			
Paul	Hong			Mayo Clinic Comprehensive Cancer Center, Phoenix, Arizona, USA			
Archit	Gupta			Department of GI Surgery, Medanta the Medicity, Gurugram, India			
Congde	MartinXu			Department of Surgery, Munich, Germany			
Georgios	Konstantoudakis			Nicosia General Hospital, Cyprus			
Thalis	Christophides			Nicosia General Hospital, Cyprus			
Matthew	McGuinness			North Shore Hospital, Auckland, New Zealand			
Eleanor	Spurring			Nottingham University Hospitals NHS Trust, UK			
Yasuhito	Iwao			Ohta Nishinouchi Hospital, Kōriyama, Japan			

***First Name and Middle Initial(s)**	***Last Name**	***Suffix (eg, Jr, III)**	Academic Degrees	Institution	Location (city, state/province, country)	Role or Contribution, eg, chair, principal investigator	Group (if more than 1 Group listed in the byline) and/or Subgroup (eg, Steering Committee)
Sheraz	Yaqub			Department of HPB Surgery, Oslo University Hospital, Norway			
Martin	Pirklc			Department of Hepatopancreatobiliary Surgery, Oxford, UK			
Ruhi	Vaitha			Department of Hepatopancreatobiliary Surgery, Oxford, UK			
Riccardo	Pellegrini			Hepatopancreatobiliary and Liver Transplant Surgery, University of Padova, Italy			
Giampaolo	Perri			Hepatopancreatobiliary and Liver Transplant Surgery, University of Padova, Italy			
Navneet	Tiwari			Royal Free London, UK			
Andrea	Sheel			Royal Liverpool Hospital, UK			
Amy	Sheen			Royal North Shore Hospital, Sydney, Australia			
Krishna	Kotecha			Royal North Shore Hospital, Sydney, Australia			
Daisy	Evans			Royal Stoke University Hospital, UK			
Omid	Ghamarnejad			Klinikum Saarbrücken, Germany			
Gregor A.	Stavrou			Klinikum Saarbrücken, Germany			
Rikhilroy	Patel			Southampton NHS Foundation Trust, Southampton, UK			
Dimitrios	Karavias			Southampton NHS Foundation Trust, Southampton, UK			

***First Name and Middle Initial(s)**	***Last Name**	***Suffix (eg, Jr, III)**	Academic Degrees	Institution	Location (city, state/province, country)	Role or Contribution, eg, chair, principal investigator	Group (if more than 1 Group listed in the byline) and/or Subgroup (eg, Steering Committee)
Mihaela	Misca			Spitalul Clinic, Bucharest, Romania			
John	Hammond			St Vincent Hospital, Dublin, Ireland			
Marcus Thomas Thor	Roalso			Department of Gastrointestinal Surgery, HPB Unit, Stavanger University Hospital, Norway			
Faiza	Soomro			St. James's University Hospital, The Leeds Teaching Hospitals, UK			
Asif	Halimi			Umeå, Sweden			
Timothy	Kendall			Department of Pathology, Royal Infirmary of Edinburgh, UK			
Visad	Patel			Department of Pathology, Royal Infirmary of Edinburgh, UK			
Dunja	Stankić			First Surgical Clinic, University Clinical Center of Serbia			
Nikica	Grubor			First Surgical Clinic, University Clinical Center of Serbia			
Pieter	Dries			University Hospital Gent, Belgium			
Charlotte	Hein			University Medical Center Schleswig-Holstein, Germany			
Julia	Betram			University Medical Center Schleswig-Holstein, Campus Luebeck, Germany			
Rosetta	Jacob			University of Cincinnati, USA			
Gregory	Wilson			University of Cincinnati, USA			

***First Name and Middle Initial(s)**	***Last Name**	***Suffix (eg, Jr, III)**	Academic Degrees	Institution	Location (city, state/province, country)	Role or Contribution, eg, chair, principal investigator	Group (if more than 1 Group listed in the byline) and/or Subgroup (eg, Steering Committee)
Souheil	Redas			Department of Surgical Sciences, Uppsala University, Sweden			
Aiste	Gulla			Vilnius University Hospital, Lithuania			
Augustas	Poškus			Vilnius University Hospital, Lithuania			
Paula	Mosert			Vilnius University Hospital, Lithuania			
Ugnė	Šilinskaitė			Vilnius University Hospital, Lithuania			
Chang Moo	Kang			Yonsei University, South Korea			
Takaaki	Furukawa			Department of Hepato-Biliary-Pancreatic Medicine, Cancer Institute Hospital, Japanese Foundation for Cancer Research			
Yuki	Hirose			Department of Hepato-Biliary-Pancreatic Medicine, Cancer Institute Hospital, Japanese Foundation for Cancer Research			
Lingdi	Yin			Pancreas Centre, The First Affiliated Hospital with Nanjing Medical University, Nanjing, China			
Xue	Liu			Pancreas Centre, The First Affiliated Hospital with Nanjing Medical University, Nanjing, China			

***First Name and Middle Initial(s)**	***Last Name**	***Suffix (eg, Jr, III)**	Academic Degrees	Institution	Location (city, state/province, country)	Role or Contribution, eg, chair, principal investigator	Group (if more than 1 Group listed in the byline) and/or Subgroup (eg, Steering Committee)
Lars	Standaert			Department for General and HPB Surgery and Liver Transplantation, Ghent University Hospital, Belgium			
Tegan	Kay			Peter MacCallum Cancer Centre, Melbourne, Australia			
Kenji	Nishida			Division of Pathology, The Cancer Institute of Japanese Foundation for Cancer Research			
Vivek	Peddakota			HPB unit, freeman hospital, Newcastle, UK			
