# Supplementary material for: Contemporary Adjuvant Chemotherapy for Intraductal Papillary Mucinous Neoplasms
Source: JAMA Netw Open. 2026 Mar 27;9(3):e263688. doi: 10.1001/jamanetworkopen.2026.3688 (PMC13032148; doi:10.1001/jamanetworkopen.2026.3688)
Supplement: Supplement 3. — Data Sharing Statement [file jamanetwopen-e263688-s003.pdf]

Data Sharing Statement

Lucocq. Contemporary Adjuvant Chemotherapy for Intraductal Papillary Mucinous Neoplasms. JAMA Netw Open. Published online March 27, 2026. doi:10.1001/jamanetworkopen.2026.3688

Data
Data available: No

Additional Information
Explanation for why data not available: We have multiple data sharing agreements with collaborators for this study and will make them available if needed
